# Efficacy of different nasal irrigation treatments versus placebo in allergic rhinitis: a systematic review and network meta-analysis

**DOI:** 10.3389/fphar.2025.1670372

**Published:** 2025-11-10

**Authors:** Qidi Hu, Lili Kong, Yi Zhou, Wen Shen, Yao Sun, Jing Deng

**Affiliations:** 1 Department of Otolaryngology, Jiaxing University Master Degree Cultivation Base, Zhejiang Chinese Medical University, Hangzhou, Zhejiang, China; 2 Department of Otolaryngology, The First Hospital of Jiaxing, Jiaxing, China

**Keywords:** allergic rhinitis, nasal lavage, network meta-analysis, systematic review, botanicaldrug

## Abstract

**Purpose:**

Allergic rhinitis (AR) is a globally prevalent disease, and nasal irrigation is one of its effective treatments. This study aims to compare the efficacy and effectiveness of different nasal irrigation treatments on AR patients’ nasal symptoms and quality of life (QoL).

**Methods:**

Studies on AR patients using different nasal irrigation treatments were searched from Cochrane, Embase, Pubmed, Web of Science, China National Knowledge Infrastructure (CNKI), VIP Database, and China Biology Medicine disc (CBM) up to 29 March 2025. The quality of the included studies was independently assessed using the NIH quality evaluation tool. The primary outcomes included relevant scale scores from the Total Nasal Symptom Score (TNSS), and secondary outcomes included those from the Rhinoconjuctivitis Quality of Life Questionnaire (RQLQ). Random-effects or fixed-effects models were selected for network meta-analysis, and mean difference (MD) was calculated with a 95% credibility interval (CrI). Surface under the cumulative ranking curve (SUCRA) was employed to rank various interventions. R 4.4.1 was used for statistical analysis.

**Results:**

23 studies involving 3,584 patients were identified. The results of the network meta-analysis showed that compared with the placebo, multiple nasal irrigating agents were more significantly efficacious and effective. In terms of alleviating nasal symptoms (a lower score indicated better effectiveness), resveratrol was the most efficacious [MD: −7.7, 95% CrI (−14.0, −1.1)] (SUCRA = 92.08%), and budesonide also showed significant efficacy and effectiveness [MD: −5.6, 95% CrI (−10.0, −0.99)] (SUCRA = 83.39%). In terms of improving QoL (a lower score indicated better efficacy), cinnamon bark was the most efficacious [MD: −1.3, 95% CrI (−1.6, −0.96)](SUCRA = 95.78%). In addition, hydrogen-rich water [MD: −1.2, 95% CrI (−2.4, 0.10)](SUCRA = 82.58%) and fluticasone [MD: −0.83, 95% CrI (−0.94, −0.71)](SUCRA = 81.49%) also showed significant differences from the placebo.

**Conclusion:**

Resveratrol is the most efficacious in relieving nasal discomfort, and cinnamon bark performs best in optimizing QoL. The results of this study provide scientific evidence for the use of botanical drugs (such as resveratrol and cinnamon) in nasal irrigation for the treatment of AR, offering new options for this disease. This is conducive to promoting the application and verification of some traditional drugs within the framework of modern medicine.

**Systematic Review Registration:**

identifier CRD 420251054166.

## 1 Introduction

Allergic rhinitis (AR) is caused by immunoglobulin E (IgE) -mediated inflammation of the nasal mucosa due to allergen exposure (Wise et al., 2018). It affects almost all age groups, while its prevalence varies according to populations and regions and rapidly increases in developing countries (Ha et al., 2020; Chong and Chew, 2018). When an allergen is inhaled and adheres to the nasal mucosa, it will stimulate the production of IgE in a sensitized immune system, resulting in swelling and hyperreactivity of the nasal mucosa (Bousquet et al., 2008; London and Ramanathan, 2017; Toppila-Salmi et al., 2015). Major symptoms include runny, stuffy nose, sneezing, and itching (Min, 2010), which may influence other comorbidities (including sinusitis and asthma), further impairing patients’ quality of life (QoL), such as their social life, academic performance, and professional behaviour (Bousquet et al., 2006; Yorgancıoğlu et al., 2012). For teenagers, the prolonged impact on QoL can have significant negative impacts on physical and psychological health (Small et al., 2018).

Current clinical treatments mainly include intranasal corticosteroids (INCS), non-sedative antihistamines (AH), and decongestants (Patel et al., 2020). However, patients often report inadequate symptom relief or have difficulty in adherence (Wheatley and Togias, 2015). On the other hand, biological agents targeting specific targets (such as monoclonal antibodies) are effective in the long term, but they are very expensive and still have potential side effects, such as sedation and irritation of the nasal mucosa. In contrast, nasal irrigation is recommended for treating AR (Luo et al., 2024). Although patients can easily master the usage of nasal irrigation after health education (Hermelingmeier et al., 2012), its effectiveness may be limited in patients with persistent and severe symptoms. Therefore, researchers have explored the addition of various pharmaceutical ingredients, such as certain plant metabolites and corticosteroids, to nasal rinses to enhance their efficacy. Preliminary studies suggest that this approach may help relieve related symptoms and improve QoL in patients with AR. (Kanjanawasee et al., 2018). As far as is concerned, there are no reliable systematic reviews comparing the efficacy of different single active ingredients (e.g., corticosteroids, plant metabolites.) added to nasal rinses for the treatment of AR. Therefore, to comprehensively assess the efficacy and effectiveness of different interventions compared with placebo in improving the primary outcome (Total Nasal Symptom Score (TNSS)) and secondary outcome (Rhinoconjunctivitis Quality of Life Questionnaire (RQLQ)), we conducted a systematic review and network meta-analysis. This methodology integrates direct and indirect evidence and allows for comprehensive ranking and comparison of multiple interventions even in the absence of head-to-head comparative trials.

This study aimed to compare the efficacy and effectiveness of different active ingredients added to nasal rinses compared with placebo on nasal symptoms and QoL in adults with AR using a systematic review and network meta-analysis.

## 2 Methods

This systematic review and network meta-analysis was conducted according to Preferred Reporting Items for Meta-Analyses for Systematic reviews and Meta-Analyses (PRISMA) (Hutton et al., 2015). This study has been registered with the PROSPERO International Prospective Register of Systematic Reviews (registration number CRD 420251054166).

### 2.1 Search strategy

We searched Cochrane, Embase, Pubmed, Web of Science, China National Knowledge Infrastructure (CNKI), VIP Database, and China Biology Medicine disc (CBM) up to 29 March 2025. Main search terms were “Rhinitis and Allergic”, “Nasal”, and “Rinse or Irriga or Lavage or Flush or Spray or Washing”. The complete search strategy for all databases, including detailed search steps, search terms, and the number of articles obtained, is provided in Supplementary Table S1 to ensure transparency and reproducibility of the search process. No language limitations were adopted, but non-English studies should provide English abstracts.

### 2.2 Study selection

All studies were managed in EndNote 21. After removing duplicates, two researchers (Qidi Hu and Lili Kong) examined the titles and abstracts to select relevant studies, the full texts of which were then downloaded. Two reviewers screened separately, and a third person summarized their results. In the initial screening, studies that met at least the patient characteristics and interventions were selected, and the rest were excluded.

### 2.3 Inclusion and exclusion criteria

#### 2.3.1 Inclusion criteria

1. The study population should be patients diagnosed with AR, at least 18 years of age; 2. The intervention and control should include nasal rinses or sprays containing any active pharmaceutical ingredients (e.g., corticosteroids, antihistamines, herbal extracts); 3. Studies that provided extractable data on efficacy or effectiveness in relevant diseases; 4. The type of literature was a randomized controlled study or a cohort study. 5. The primary outcome was TNSS, which was typically used to assess four core symptoms, including runny nose, nasal congestion, nasal itching, and sneezing. The TNSS score ranged from 0 (no symptoms) to 3 (severe symptoms). A higher score indicated more severe symptoms. The secondary outcome was RQLQ. The RQLQ tool covered 28 questions across 7 domains, evaluating the impact of allergic symptoms (activity limitation, sleep problems, nasal symptoms, eye symptoms, non-nasal/non-eye symptoms, practical problems, and emotional function). Participants were asked to answer each question on a 7-point scale ranging from 0 (no impairment at all) to 6 (severe impairment), and to recall the degree of distress they experienced with allergic symptoms in the previous week. A higher RQLQ score indicated worse QoL.

#### 2.3.2 Exclusion criteria

1. Studies involving non-adults with unclear diagnostic populations; 2. Unclear interventions or controls (The route of administration of the intervention was not nasal (e.g., oral or intravenous), or the description of the intervention (rinse or spray) in the studies was incomplete, so that key information, such as its specific ingredients, cannot be determined); 3. Unavailable full-text or outcome data; 4. Not randomized controlled or cohort studies.

### 2.4 Data extraction

Two reviewers (Qidi Hu and Lili Kong) reviewed and extracted data from the selected studies. To ensure data accuracy, any inconsistencies that arose during cross-checking of the extracted results were first reviewed by two researchers and discussed. If any disagreement remained after discussion, a third person would adjudicate. The following data were collected: first author, year of publication, nationality, sample size, age, intervention, control, type of study, and outcome indicators (TNSS and RQLQ).

We obtained the means and standard deviations (SD) for changes from baseline to post-intervention as the major data source for this analysis. If no SD was provided, standard error, 95% confidence interval (CI), polar deviation, and quartiles were used to calculate it. When the interquartile range (IQR) was given, it was used as the mean and IQR/1.135 as the standard deviation (Hozo et al., 2005). If the Min-Max median was present in an eligible article, the data were not considered for statistical analysis.

### 2.5 Description of Chinese herbal compound intervention drugs

All botanical drugs evaluated in this network meta-analysis are clearly defined in Supplementary Table S2. The botanical identities of the constituent species were verified using Kew Science (http://mpns.kew.org/mpns-portal), and the identities of certain medicinal animals were cross-referenced with the GBIF database (https://www.gbif.org/). As shown in Supplementary Table S2, the details of each botanical drug was specified, including the full and valid species, constituent herbs, family, authority, verification source, pharmacopoeial drug name, and type of extract.

The botanical drugs involved in this study were as follows. Resveratrol was extracted from *Reynoutria japonica* Houtt. (Polygonaceae; Polygoni Cuspidati Rhizoma et Radix). Cinnamon bark was extracted from *Cinnamomum verum* J. Presl (Lauraceae; Cinnamomi Cortex). Nose clearing is composed of *Houttuynia cordata* Thunb. (Saururaceae; Houttuyniae Herba), *Scutellaria baicalensis* Georgi (Lamiaceae; Scutellariae Radix), *Nepeta tenuifolia* Benth. (Lamiaceae; Schizonepetae Herba Carbonisata), *Xanthium strumarium* L. (Asteraceae; Xanthii Fructus), *Conioselinum anthriscoides ‘Chuanxiong’* (Apiaceae; Chuanxiong Rhizoma), *Acorus verus* (L.) Raf. (Acoraceae; Acori Tatarinowii Rhizoma), and *Poria cocos* F.A.Wolf. E-Qi is composed of *N. tenuifolia* Benth. (Lamiaceae; Schizonepetae Herba Carbonisata), *Saposhnikovia divaricata* (Turcz. ex Ledeb.) Schischk. (Apiaceae; Saposhnikoviae Radix), *Magnolia biondii* Pamp. (Magnoliaceae; Magnoliae Flos), *Mentha canadensis* L. (Lamiaceae; Menthae Haplocalycis Herba), *Angelica dahurica* (Hoffm.) Benth. and Hook. f. ex Franch. and Sav. (Apiaceae; Angelicae Dahuricae Radix), *Vincetoxicum mukdenense* Kitag. (Apocynaceae; Cynanchi Paniculati Radix et Rhizoma), *Centipeda minima* (L.) A. Braun and Asch. (Asteraceae; Centipedae Herba), *Astragalus mongholicus* Bunge (Fabaceae; Astragali Radix), *Xanthium strumarium* L. (Asteraceae; Xanthii Fructus), and *Punica granatum* L. (Lythraceae; Granati Pericarpium). Xanthium was extracted from *Xanthium strumarium* L. (Asteraceae; Xanthii Fructus).

### 2.6 Risk of bias assessments

Two independent reviewers assessed the risk of bias in the selected studies using the NIH scale (https://www.nhlbi.nih.gov/health-topics/study-quality-assessment-tools), and a third reviewer summarized their results. Since the types of study were either randomized controlled or cohort studies, more than one kind of NIH scale was adopted. The NIH scale for randomized controlled studies included multiple categories, such as randomization, blinding, sample size assessment, interventions, outcome measures, statistical analyses, bias control, and outcome reporting. The scale for cohort studies included study design, sample selection, exposure assessment, outcome measures, follow-up time, confounding controls, statistical analysis, and reporting of results. These categories aimed to fully assess the scientific validity and reliability of randomized controlled and cohort studies, so as to guarantee the validity and accuracy of this study. Meanwhile, only studies with a low risk of bias (defined as a score greater than 10) were included in the main network meta-analysis to ensure the reliability and accuracy of this study.

### 2.7 Data synthesis and analysis

R version 4.4.1 and GeMTC were used to construct the network meta-analysis model. All data analyses were performed based on a Bayesian random-effects model, using the “GeMTC” and “rjags” packages of R software. Markov chain Monte Carlo (MCMC) methods were used to simulate parameter posterior distributions, providing probability distributions for each effect size estimate. We set a total number of parameter tuning iterations to 25,000, performed 250,000 simulations, and adopted a 10-fold sampling factor to generate the Bayesian statistical model for the network meta-analysis. Model consistency was verified using both consistency and inconsistency models. When closed loops were observed in the network diagram, a node-splitting method was used to assess local inconsistencies between direct and indirect evidence. Model convergence was assessed using the Brooks-Gelman-Rubin diagnostic tool as well as trajectory and density plots. Effect sizes were calculated using the Bayesian random-effects model with mean difference (SMD) and 95% credibility intervals (95% CrIs). Comparisons of all interventions were presented using forest plots and summary tables. Statistical significance was determined when 95% CrIs did not include zero. Different interventions were ranked and hierarchically stratified by calculating the surface under the cumulative ranking curve (SUCRA). SUCRA values ranged from 0% to 100%. A higher value indicated a greater likelihood of being the most effective intervention measure. A network diagram was used to visualize the relative relationships among the different interventions in this network meta-analysis, where nodes represented different interventions, and edges corresponded to direct comparisons between interventions.

### 2.8 Sensitivity analysis and publication bias

To assess the robustness of our findings, a sensitivity analysis was conducted by excluding the studies one by one. Analytical variables included comparisons of statistical results of different methodologies (fixed-effect and random-effect models). Studies with low quality scores were excluded from the analysis, and changes in the results before and after these exclusions were compared. The publication bias was assessed using Egger’s and Begger’s test. A P < 0.5 indicated the presence of publication bias; otherwise, no publication bias was considered.

## 3 Results

### 3.1 Study selection

**FIGURE 1:**
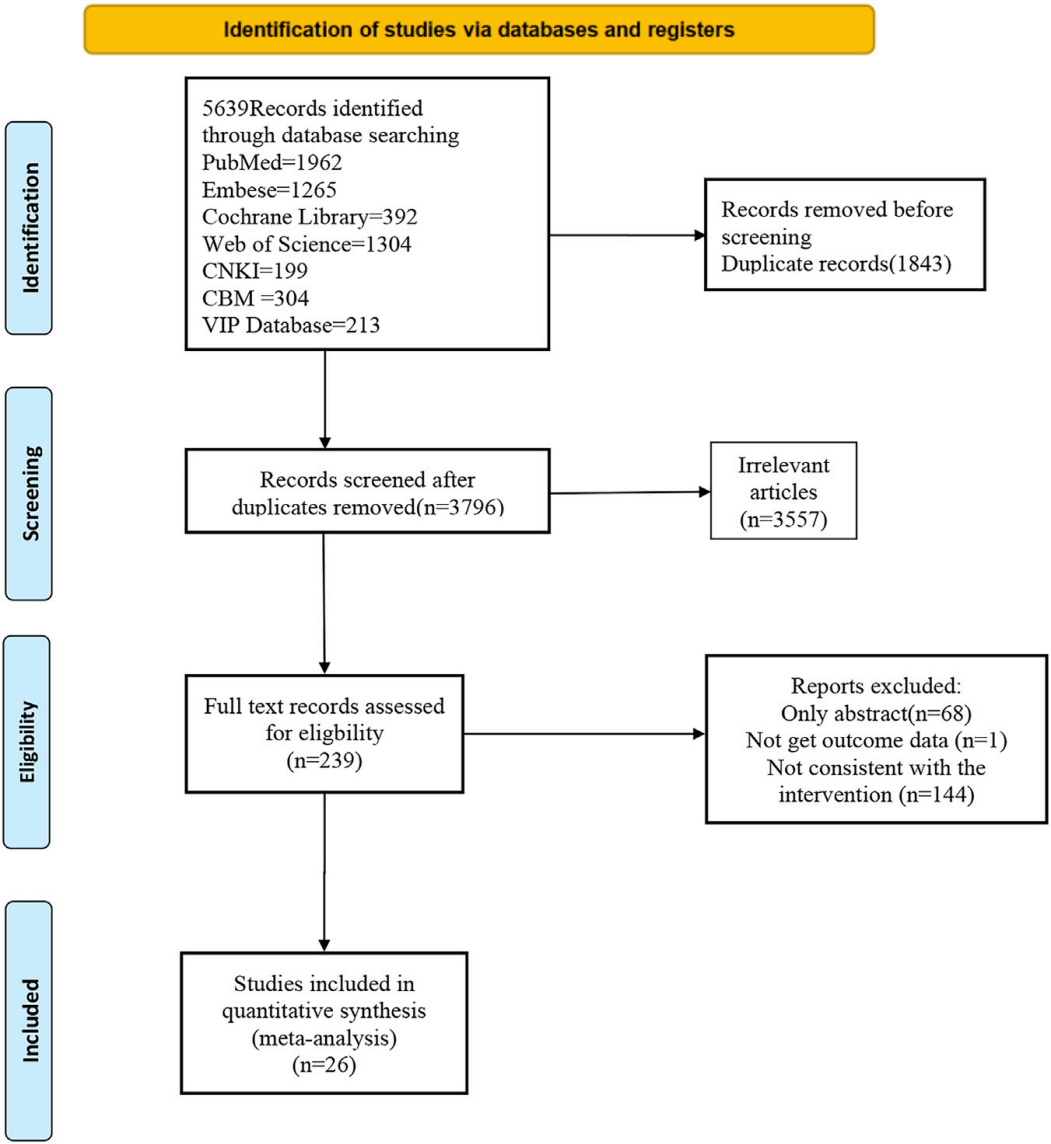
A flow chart for new systematic review retrieval and selection.

A total of 5,639 articles were identified from databases. 1843 of those were duplicates, and 3,557 were excluded based on the inclusion and exclusion criteria. Among the remaining 239, the full texts of 68 were unavailable. After reading the full texts, 144 were excluded due to ineligible interventions, and 1 for the absence of an intervention outcome. In the end, 26 studies published up to 29 March 2025 were included. The complete selection process is shown in the PRISMA flowchart in Figure 1.

### 3.2 Basic characteristics of the included studies

The 26 eligible studies (Kim et al., 2022; Magen et al., 2006; Wu et al., 2014; Zhang, 2023; Coyte, 1990; Jin et al., 2022; Jahan and Yong, 2018; Zhang and Yao, 2013; Wu et al., 2012; Sansila et al., 2020; Wang, 2016; Ratner et al., 2006; Singh et al., 2016; Berkowitz et al., 1999; Jin et al., 2018; Gendeh et al., 2024; Ciprandi et al., 2003; Lv et al., 2018; Hampel et al., 2006; Ellis et al., 2016; Rimmer et al., 2012; Badorrek et al., 2015; Dykewicz et al., 2003; Yamada et al., 2012; Steels et al., 2019; Long, 2013) encompassed 3,872 patients. The basic characteristics of the included studies are described in Table 1. Eleven interventions were reported: fluticasone (FLU), budesonide (BUD), BUD + normal saline (NS), hydrogen-rich saline (HRS), BUD + hypertonic saline (HS), HS, ciclesonide (CIC), mometasone furoate (MF), olopatadine (OLO), cinnamon bark (CB), HOCl, mometasone furoate + NS, nose clearing (NC), E-Qi, xanthium (XAN), resveratrol (RESV), and S0597 (a novel intranasal glucocorticosteroid). The control group received placebo. In terms of the types of studies, there were 25 randomized controlled studies and 1 cohort study. The age of the sample population ranged from 19 to 60 years.

**TABLE 1:** Study characteristics of randomized controlled trials/cohort studies comparing different nasal irrigants with placebo.

Study	Country	Diagnosis	Patients	Age	Intervention	Intervention patients	Control patients	Outcome	Time of treatment	Type
Ho Chan Kim 2022	Korea	AR	114	31.30 ± 13.48	HOCl vs. NS	55	59	TNSS	4 weeks	RCT
E Magen 2006	Israel	AR	68	46.4 ± 11.04	FLU vs. NS	34	34	RQLQ	8 weeks	RCT
Minghai Wu 2014	China	AR	61	NA	BUD vs. BUD + NS vs. NS	50	21	RQLQ	3 months	RCT
Minxiong Zhang 2023	China	AR	104	39.2 ± 7.26	HRS vs. NS	52	52	RQLQ	4 weeks	RCT
John T. Given 2010	United States	AR	315	38.69 ± 14.64	FLU vs. Placebo	160	155	RQLQ	4 weeks	RCT
Ling Jin 2022	China	AR	54	41.49 ± 15.51	HRS vs. NS	29	25	TNSS	4 weeks	RCT
JIAHAN·Hazaiguli 2018	China	AR	180	33.5 ± 8.15	BUD vs. BUD + HS vs. HS	120	60	RQLQ	4 weeks	Cohort
Dongxia Zhang 2013	China	AR	120	36.57 ± 5.93	NC vs. NS	60	60	TNSS	4 weeks	RCT
Dongqiu Long 2013	China	AR	124	35.2 ± 6.9	E-Qi vs. NS	62	62	TNSS	3 weeks	RCT
Feihu Wu 2012	China	AR	80	NA	E-Qi vs. NS	40	40	TNSS	3 weeks	RCT
Kittiyaporn Sansila 2020	Thailand	AR	78	36.85 ± 11.26	HS vs. NS	35	43	TNSS	4 weeks	RCT
Lili Wang 2016	China	AR	80	36.70 ± 4.82	XAN vs. NS	40	40	TNSS	10 days	RCT
Paul H. Ratner 2006	United States	AR	327	40 ± 14.5	CIC vs. Placebo	164	163	RQLQ	2 weeks	RCT
Roohie Singh 2016	India	AR	60	NA	HS vs. NS	30	30	TNSS	2 months	RCT
R. B. Berkowitz 1999	United States	AR	235	33.06 ± 11.63	MF vs. Placebo	119	116	TNSS	12 h	RCT
Ling Jin 2018	China	AR	20	38.95 ± 11	HRS vs. NS	20	20	RQLQ	4 weeks	RCT
Hardip S. Gendeh 2024	Malaysia	AR	163	31.23 ± 8.29	MF vs. NS	110	53	RQLQ	30 days	RCT
Giorgio Ciprandi 2003	Italy	AR	20	24.7 ± 3.25	BUD vs. NS	10	10	TNSS	2 weeks	RCT
Chunjiang Lv 2018	China	AR	151	NA	RESV vs. BUD vs. Placebo	101	50	TNSS	1 month	RCT
Frank C. Hampel 2006	United States	AR	675	38.87 ± 14.73	OLO vs. Placebo	451	224	RQLQ	2 weeks	RCT
Anne K. Ellis 2016	Canada	AR	222	39 ± 10.4	S0597 vs. Placebo	166	56	TNSS	2 weeks	RCT
Janet Rimmer 2012	Sweden	AR	19	40.1 ± 12.8	FLU vs. NS	19	19	RQLQ	6 weeks	RCT
Philipp Badorrek 2015	Germany	AR	159	37.8 ± 11.1	S0597 vs. Placebo	119	40	TNSS	15 days	RCT
Mark S. Dykewicz 2003	United States	AR	241	34 ± 14.34	FLU vs. Placebo	122	119	TNSS	4 weeks	RCT
Takechiyo Yamada 2012	Japan	AR	56	22.6 ± 6.5	MF vs. Placebo	56	56	TNSS、RQLQ	2 weeks	RCT
Eleanor Steels 2019	Australia	AR	60	43.25 ± 14.39	CB vs. NS	30	30	RQLQ	1 week	RCT

NS, normal saline; HS, hypertonic saline; HRS, Hydrogen-rich water; NC, nose clearing; XAN, xanthium; CIC, ciclesonide; RESV, resveratrol; FLU, fluticasone; BUD, budesonide; MF, mometasone furoate; OLO, olopatadine; CB, cinnamon bark; TNSS, total nasal symptom score; RQLQ, rhinoconjunctivitis quality of life questionnaire.

### 3.3 Risk of bias assessments

The risk of bias assessment results are shown in Table 2. Seventeen studies scored 12 points or more, indicating a high research quality; six studies scored more than 10, indicating a good research quality; three studies scored 10 or less, indicating a high risk of bias. To ensure the robustness of the primary analysis, the three studies with a high risk of bias were excluded, and the network meta-analyses for TNSS and RQLQ were conducted using the remaining 23 studies. Among them, randomized controlled studies had a higher quality score, while cohort studies had a lower score. The common risk of randomized controlled studies stemmed from the incomplete blinding and allocation concealment of participants and personnel. For cohort studies, the risk of bias was from the selection of sample size, the relationship between exposure level and outcome, whether multiple measurements were taken, and the blinding of participants and personnel.

**TABLE 2:** The risk of bias assessment.

NIH quality assessment of controlled intervention studies															
NO.	1	2	3	4	5	6	7	8	9	10	11	12	13	14	Overall
Ho Chan Kim 2022	Y	N	N	Y	Y	Y	Y	Y	Y	Y	Y	Y	Y	Y	12
E Magen 2006	Y	N	N	Y	N	Y	Y	Y	Y	Y	Y	Y	Y	Y	11
Minghai Wu 2014	Y	N	N	Y	N	Y	Y	Y	Y	Y	Y	Y	Y	Y	11
Minxiong Zhang 2023	Y	N	N	Y	N	Y	Y	Y	Y	Y	Y	Y	N	Y	10
John T. Given 2010	Y	N	N	Y	Y	Y	Y	Y	Y	Y	Y	Y	Y	Y	12
Dongqiu Long 2013	Y	N	N	Y	N	Y	Y	Y	Y	Y	Y	Y	N	Y	10
Ling Jin 2022	Y	Y	N	Y	Y	Y	Y	Y	Y	Y	Y	Y	Y	Y	13
Dongxia Zhang 2013	Y	N	N	Y	N	Y	Y	Y	Y	Y	Y	Y	Y	Y	11
Feihu Wu 2012	Y	N	N	Y	N	Y	Y	Y	Y	Y	Y	Y	Y	Y	11
Kittiyaporn Sansila 2020	Y	Y	Y	Y	Y	Y	Y	Y	Y	Y	Y	Y	Y	Y	14
Lili Wang 2016	Y	N	N	Y	N	Y	Y	Y	Y	Y	Y	Y	Y	Y	11
Paul H. Ratner 2006	Y	Y	N	Y	Y	Y	Y	Y	Y	Y	Y	Y	Y	Y	13
Roohie Singh 2016	Y	N	N	Y	N	Y	Y	Y	Y	Y	Y	N	Y	Y	10
R. B. Berkowitz 1999	Y	Y	N	Y	Y	Y	Y	Y	Y	Y	Y	Y	Y	Y	13
Ling Jin 2018	Y	N	Y	Y	Y	Y	Y	Y	Y	Y	Y	Y	Y	Y	13
Hardip S. Gendeh 2024	Y	Y	Y	Y	Y	Y	Y	Y	Y	Y	Y	Y	Y	Y	14
Giorgio Ciprandi 2003	Y	N	N	Y	Y	Y	Y	Y	Y	Y	Y	Y	Y	Y	12
Chunjiang Lv 2018	Y	N	N	Y	Y	Y	Y	Y	Y	Y	Y	Y	Y	Y	12
Frank C. Hampel 2006	Y	N	N	Y	Y	Y	Y	Y	Y	Y	Y	Y	Y	Y	12
Anne K. Ellis 2016	Y	N	N	Y	Y	Y	Y	Y	Y	Y	Y	Y	Y	Y	12
Janet Rimmer 2012	Y	Y	N	Y	Y	Y	Y	Y	Y	Y	Y	N	Y	Y	12
Philipp Badorrek 2015	Y	Y	N	Y	Y	Y	Y	Y	Y	Y	Y	Y	Y	Y	13
Mark S. Dykewicz 2003	Y	N	N	Y	Y	Y	Y	Y	Y	Y	Y	N	Y	Y	11
Takechiyo Yamada 2012	Y	N	N	Y	Y	Y	Y	Y	Y	Y	Y	Y	Y	Y	12
Eleanor Steels 2019	Y	Y	Y	Y	Y	Y	Y	Y	Y	Y	Y	Y	Y	Y	14
NIH Quality Assessment Tool for Observational Cohort and Cross-Sectional Studies of Controlled Intervention Studies															
NO.	1	2	3	4	5	6	7	8	9	10	11	12	13	14	Overall
JIAHAN·Hazaiguli 2018	Y	Y	Y	Y	N	Y	Y	Y	Y	Y	Y	Y	Y	Y	13

1Was the study described as randomized, a randomized trial, a randomized clinical trial, or an RCT?

2Was the method of randomization adequate (i.e., use of randomly generated assignment)?

3Was the treatment allocation concealed (so that assignments could not be predicted)?

4Were study participants and providers blinded to treatment group assignment?

5Were the people assessing the outcomes blinded to the participants’ group assignments?

6Were the groups similar at baseline on important characteristics that could affect outcomes (e.g., demographics, risk factors, co-morbid conditions)?

7Was the overall drop-out rate from the study at endpoint 20% or lower of the number allocated to treatment?

8Was the differential drop-out rate (between treatment groups) at endpoint 15 percentage points or lower?

9Was there high adherence to the intervention protocols for each treatment group?

10Were other interventions avoided or similar in the groups (e.g., similar background treatments)?

11Were outcomes assessed using valid and reliable measures, implemented consistently across all study participants?

12Did the authors report that the sample size was sufficiently large to be able to detect a difference in the main outcome between groups with at least 80% power?

13Were outcomes reported or subgroups analyzed prespecified (i.e., identified before analyses were conducted)?

14Were all randomized participants analyzed in the group to which they were originally assigned, i.e., did they use an intention-to-treat analysis?

### 3.4 Publication bias and sensitivity analysis

Publication bias was assessed for RQLQ and TNSS using Begg’s test and Egger’s funnel plots. The funnel plot for RQLQ was symmetrical, and Begg’s test (P = 0.71) and Egger’s test (P = 0.55) indicated no publication bias (Supplementary Figure S2). The funnel plot for TNSS was symmetrical, and Begg’s test (P = 0.06) and Egger’s test (P = 0.98) indicated no publication bias (Supplementary Figure S3).

To confirm the robustness of the results, a sensitivity analysis of RQLQ and TNSS was conducted based on the models selected and studies included (Supplementary Table S3, S4).The results of the top three effective interventions ranked by SUCRA values under the fixed-effects model were found to be consistent with those of the top three effective interventions under the random-effects model. Therefore, the results were methodologically robust, and the model used had no impact on the primary results of RQLQ. Regarding the inclusion of studies, a fixed-effect model was used to exclude studies with low quality scores, and the effects of these exclusions on the primary outcome were compared before and after exclusion. The results of the top three interventions ranked before exclusion were found to be consistent with those of the top three effective interventions after exclusion. Therefore, the studies included had no impact on the main results of RQLQ, and the results were relatively robust.

For TNSS, the results of the top three effective interventions ranked by SUCRA values in the fixed-effects model were consistent with those of the top three effective interventions in the random-effects model. Therefore, the model used had no impact on the main results of TNSS, and the results were relatively robust. Regarding the inclusion of studies, a random-effects model was used to exclude studies with low quality scores and compare the results before and after exclusion. The results of the top three interventions before exclusion were found to be consistent with those of the top three effective interventions after exclusion. Therefore, the studies included had no impact on the main results of TNSS, and the results were relatively robust. We then excluded studies with small sample sizes and compared the main results before and after exclusion. The results of the top three interventions before exclusion were consistent with those of the top three effective interventions after exclusion, further demonstrating the stability of our results.

### 3.5 Effect of different measures on nasal symptoms

**FIGURE 2:**
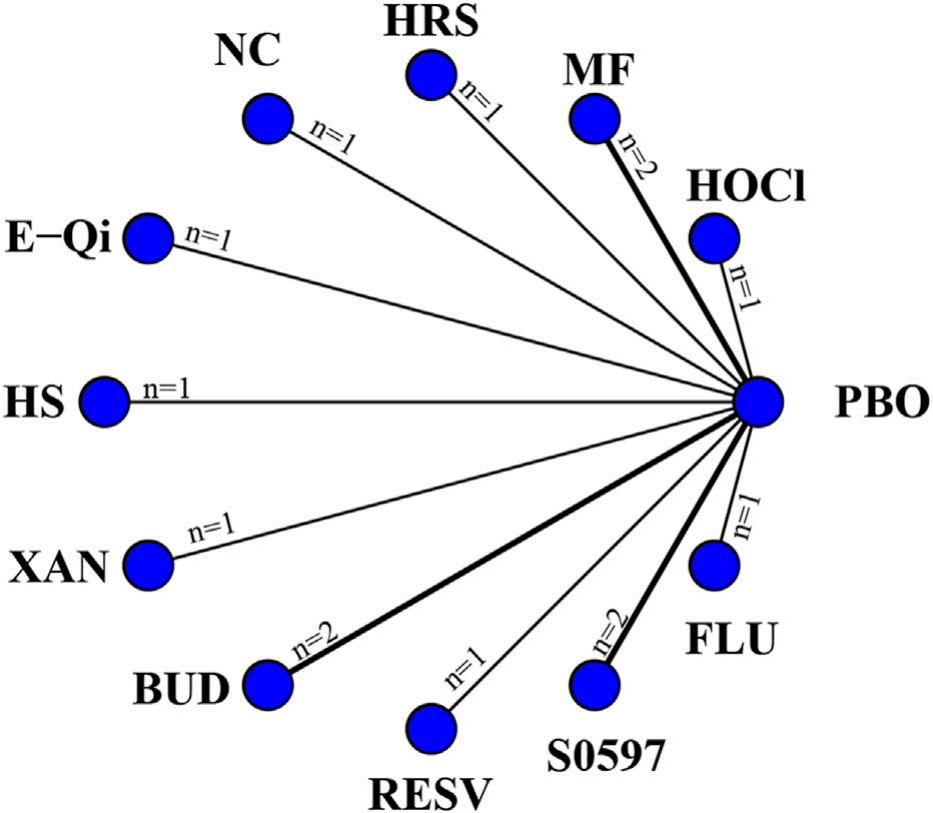
Networks of treatment comparisons for outcomes reflecting changes in the TNSS from baseline. The nodes represent the interventions. The edges represent direct comparisons between interventions, and their thickness is proportional to the number of trials examining each intervention.

**FIGURE 3:**
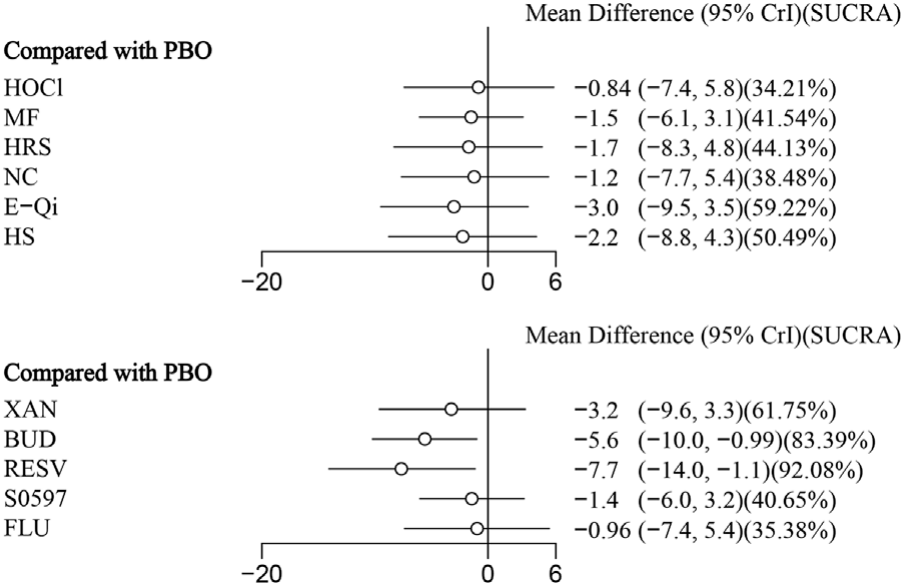
TNSS relative effect forest plot.

14 eligible studies analyzed nasal symptoms (TNSS), involving 1,670 participants. Interventions included HOCl, MF, HRS, NC, E-Qi, HS, XAN, BUD, RESV, S0597, and FLU. Among them, RESV was the most efficacious in reducing nasal symptoms [MD: 7.7,95%Crl (−14.0, −1.1)](SUCRA = 92.08%), as shown in Figure 3, followed by BUD [MD: 5.6,95%Crl (−10.0, −0.99)](SUCRA = 83.39%). The inconsistency test of the node splitting method for some subgroups was not performed because no loop was formed in the network diagram. Detailed results are shown in the network diagram (Figure 2), forest plot (Figure 3), and league table (Table 3).

**TABLE 3:** League table of improvements in nasal symptoms in patients with allergic rhinitis by different nasal irrigation formulations compared with placebo, with significant relative effects between the two interventions described in bold.

PBO											
0.84 (−5.82, 7.41)	HOCl										
1.49 (−3.09, 6.05)	0.63 (−7.34, 8.72)	MF									
1.7 (−4.79, 8.31)	0.86 (−8.47, 10.15)	0.21 (−7.77, 8.25)	HRS								
1.22 (−5.36, 7.65)	0.39 (−8.89, 9.65)	−0.27 (−8.29, 7.64)	−0.48 (−9.74, 8.61)	NC							
3.01 (−3.54, 9.47)	2.18 (−7.09, 11.32)	1.51 (−6.52, 9.54)	1.31 (−8.02, 10.42)	1.79 (−7.39, 10.94)	E-Qi						
2.23 (−4.29, 8.77)	1.4 (−7.81, 10.67)	0.75 (−7.3, 8.71)	0.54 (−8.72, 9.72)	1.02 (−8.2, 10.21)	−0.77 (−9.95, 8.59)	HS					
3.24 (−3.3, 9.61)	2.4 (−6.82, 11.54)	1.75 (−6.2, 9.65)	1.54 (−7.74, 10.59)	2.02 (−7.18, 11.19)	0.23 (−9, 9.42)	1.01 (−8.31, 10.06)	XAN				
**5.58 (0.99, 10.21)**	4.76 (−3.24, 12.81)	4.1 (−2.37, 10.54)	3.89 (−4.14, 11.95)	4.36 (−3.49, 12.44)	2.57 (−5.24, 10.65)	3.35 (−4.67, 11.41)	2.34 (−5.46, 10.33)	BUD			
**7.66 (1.14, 14.06)**	6.83 (−2.46, 16.04)	6.18 (−1.79, 14.03)	5.96 (−3.26, 15.07)	6.45 (−2.71, 15.66)	4.66 (−4.64, 13.79)	5.44 (−3.83, 14.56)	4.43 (−4.64, 13.58)	2.08 (−5.92, 9.92)	RESV		
1.43 (−3.19, 6.02)	0.6 (−7.51, 8.72)	−0.06 (−6.57, 6.45)	−0.28 (−8.31, 7.66)	0.2 (−7.78, 8.26)	−1.59 (−9.46, 6.44)	−0.82 (−8.8, 7.27)	−1.82 (−9.65, 6.22)	−4.17 (−10.7, 2.24)	−6.24 (−14.07, 1.7)	S0597	
0.96 (−5.42, 7.37)	0.12 (−8.93, 9.37)	−0.53 (−8.35, 7.39)	−0.73 (−9.9, 8.37)	−0.25 (−9.36, 8.98)	−2.05 (−11.17, 7.18)	−1.27 (−10.42, 7.82)	−2.27 (−11.23, 6.86)	−4.62 (−12.51, 3.19)	−6.71 (−15.71, 2.41)	−0.47 (−8.29, 7.35)	FLU

### 3.6 Effect of different measures on QoL

**FIGURE 4:**
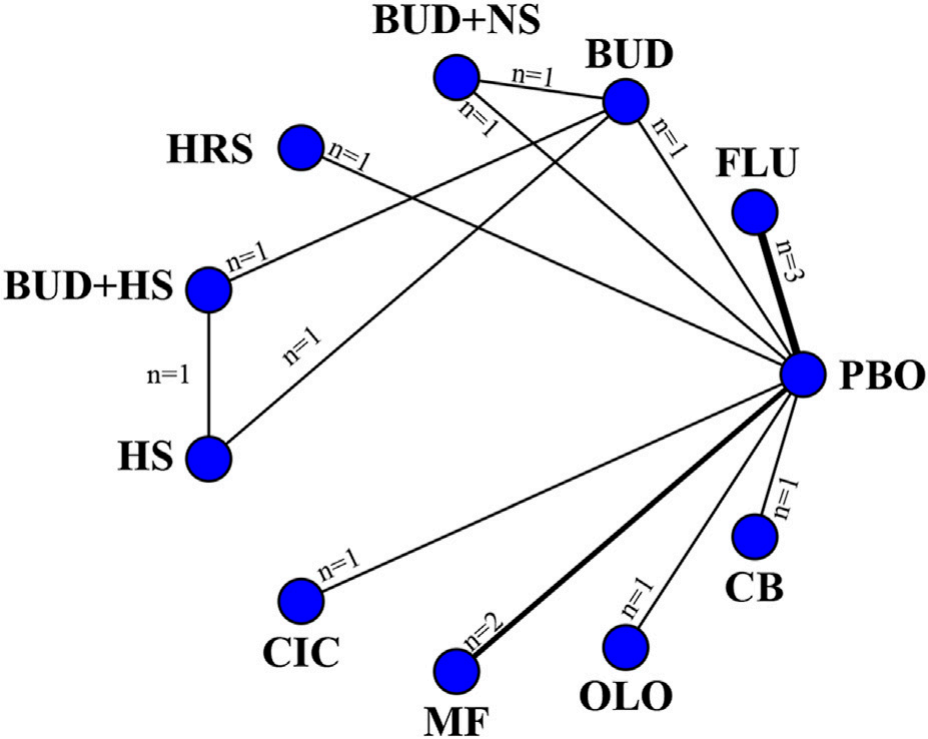
Networks of treatment comparisons for outcomes reflecting changes in the RQLQ from baseline. The nodes represent the interventions. The edges represent direct comparisons between interventions, and their thickness is proportional to the number of trials examining each intervention.

**FIGURE 5:**
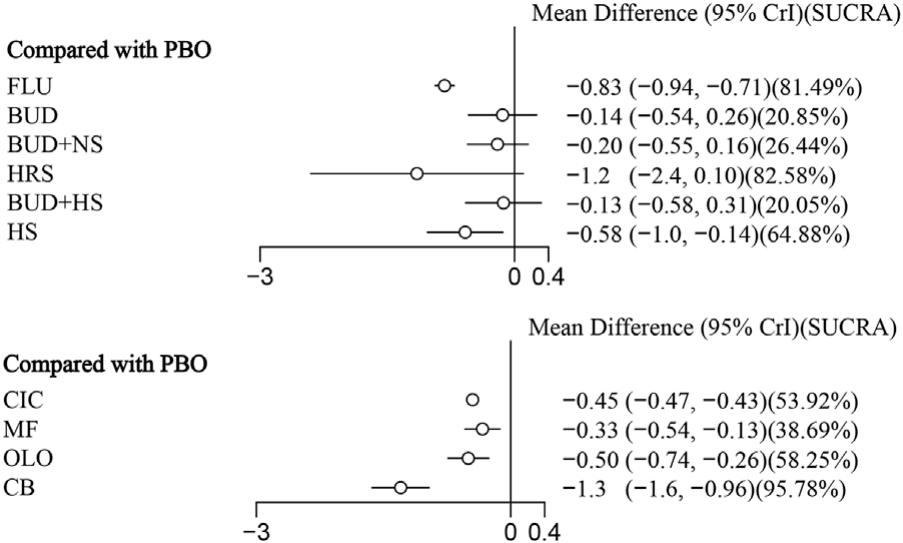
RQLQ relative effect forest plot.

11 eligible studies reported RQLQ, involving 1944 participants. Interventions included FLU, BUD, BUD + NS, HRS, BUD + HS, HS, CIC, MF, OLO, and CB. Among them, CB was the most efficacious [MD: −1.3,95%Crl (−1.6, −0.96)](SUCRA = 95.78%), followed by FLU [MD:−0.83,95%Crl (−0.94, −0.71)](SUCRA = 81.49%). The results of the inconsistency test showed that the p-values for the comparison of the different interventions were all greater than 0.05, indicating that there was no inconsistency. Detailed results are shown in the network diagram (Figure 4) and forest plot (Figure 5), league table (Table 4), and the inconsistency test results are provided in Supplementary Figure S1.

**TABLE 4:** League table of improvement in quality of life of patients with allergic rhinitis by different nasal irrigation formulations compared with placebo, with significant relative effects between the two interventions described in bold.

PBO										
**0.71 (0.15, 1.17)**	FLU									
0.14 (−0.62, 0.89)	−0.57 (−1.44, 0.38)	BUD								
0.2 (−0.54, 0.93)	−0.52 (−1.37, 0.43)	0.06 (−0.72, 0.83)	BUD + NS							
1.16 (−0.32, 2.61)	0.45 (−1.09, 2)	1.02 (−0.64, 2.65)	0.96 (−0.68, 2.59)	HRS						
0.13 (−0.89, 1.15)	−0.58 (−1.68, 0.6)	−0.01 (−0.69, 0.68)	−0.07 (−1.09, 0.97)	−1.03 (−2.8, 0.76)	BUD + HS					
0.58 (−0.44, 1.61)	−0.13 (−1.23, 1.06)	0.44 (−0.25, 1.13)	0.38 (−0.65, 1.41)	−0.58 (−2.35, 1.22)	0.45 (−0.23, 1.13)	HS				
0.45 (−0.35, 1.26)	−0.27 (−1.16, 0.74)	0.31 (−0.78, 1.42)	0.25 (−0.84, 1.35)	−0.71 (−2.36, 0.97)	0.32 (−1, 1.63)	−0.13 (−1.45, 1.17)	CIC			
0.26 (−0.39, 0.86)	−0.45 (−1.24, 0.38)	0.13 (−0.88, 1.08)	0.07 (−0.92, 1.01)	−0.89 (−2.49, 0.69)	0.13 (−1.09, 1.3)	−0.32 (−1.55, 0.86)	−0.18 (−1.24, 0.81)	MF		
0.5 (−0.34, 1.34)	−0.21 (−1.14, 0.82)	0.36 (−0.75, 1.49)	0.3 (−0.81, 1.43)	−0.66 (−2.32, 1.03)	0.37 (−0.94, 1.69)	−0.08 (−1.41, 1.24)	0.05 (−1.12, 1.21)	0.24 (−0.78, 1.31)	OLO	
**1.3 (0.43, 2.15)**	0.59 (−0.37, 1.64)	**1.16 (0.02, 2.29)**	1.1 (−0.03, 2.24)	0.14 (−1.56, 1.84)	1.17 (−0.17, 2.51)	0.72 (−0.63, 2.06)	0.85 (−0.33, 2.02)	**1.04 (0, 2.13)**	0.8 (−0.41, 2)	CB

## 4 Discussion

This systematic review and network meta-analysis included 23 studies, involving 3,584 participants and 11 various nasal rinsing treatments. It comprehensively assessed different active ingredients added to nasal rinsing solutions. The analysis demonstrated that both RESV [MD: −7.7,95%CrI(−14.0, −1.1) ] (SUCRA = 92.08%) and BUD [MD: 5.6,95%Crl (−10.0, −0.99)](SUCRA = 83.39%) significantly relieved TNSS compared to placebo. Additionally, CB [MD: −1.3,95%Crl (−1.6, −0.96)](SUCRA = 95.78%) and FLU [MD:−0.83,95%Crl (−0.94, −0.71)](SUCRA = 81.49%) showed statistically significant benefits in optimizing RQLQ scores compared to placebo.

This study found that RESV and BUD had significant efficacy in relieving nasal symptoms compared to placebo (saline-based) in AR patients. AR is triggered by exposure to allergens (e.g., pollen, dust mites) and is characterized by a complex inflammatory response. This response involves the interaction and activation of key immune cells, including eosinophils, T-cells, mast cells, and basophils (Naclerio, 1991), similar to allergic asthma. Among them, nasal mast cells and T cells are an important source of Th2 cytokines such as IL-4 and IL-13, which promote the synthesis of IgE and the production, recruitment, and activation of eosinophils (Pawankar et al., 2000). Therefore, IgE and cytokines (including IL-4) play a crucial role in the occurrence of AR (Pawankar et al., 2011). RESV is a non-flavonoid polyphenol metabolite that is widely used in traditional Chinese medicine. It is primarily derived from Polygonum cupsidatum (Reynoutria japonica Houtt.) (Hu and Li, 2019), and can also be found in multiple plants, such as grapes, berries, and peanuts. It is known for antioxidant (Xing et al., 2020) and anti-inflammatory (Chen et al., 2020) effects both *in vitro* and *in vivo*. In clinical practice, many Chinese traditional medicine treatments commonly used for the treatment of chronic inflammation contain RESV, such as Detoxification and Deflagration Granule and Compound Scutellaria Tablet (Xie and Du, 2011; Shen et al., 2017). In one study, incubation of IL-33 and IgE/antigen-stimulated RBL-2 H3 cells with RESV reduced the phosphorylation levels of p38, inhibitor of nuclear factor kappa B (IκBα), and the NF-κB subunit p65 by more than 50%, which play an important role in the pathogenesis of atopic diseases, such as AR (Xu et al., 2020; Han et al., 2015). In an experimentally induced AR rat model, Bozdemir et al. (Bozdemir et al., 2016) showed that RESV treatment reduced allergic symptoms and tissue reactions. It was also demonstrated in Chunjiang Lv 2018 that IgE and IL-4 levels in the blood of patients receiving RESV were significantly reduced, further revealing the efficacy of RESV in the treatment of AR. Similarly, the immunopathologic features of AR are related to the action of Th2-derived cytokines (Christodoulopoulos et al., 2000). Topical corticosteroids have been recognized as highly effective therapies for the treatment of AR because of their antiallergic activity, and they cause few side effects at recommended doses (Nielsen et al., 2001). BUD is the most commonly used glucocorticosteroid in the topical treatment of asthma and rhinitis and has been shown to be highly effective and safe (Snidvongs and Thanaviratananich, 2017). Relevant *in vitro* studies have reported that BUD inhibits the production of Th2 cytokines and the infiltration of inflammatory cells by activating the transcription of anti-inflammatory genes and inhibiting the transcription of pro-inflammatory genes (Mullol et al., 2009). It also significantly reduces IL-6 and IL-8 in patients with AR (Xaubet et al., 2001). Various randomized trials and meta-analyses have also demonstrated that BUD is more effective than oral antihistamines in relieving nasal obstruction, sniffling, sneezing, itchy nose, and postnasal drip (Rinne et al., 2002; Munch et al., 1983; Cai et al., 2025), but in order to shorten the time to symptom relief, BUD in combination with loratadine is more effective than hormones or antihistamines alone and improves immune function (e.g., modulation of T-lymphocyte subpopulations) (Zhang et al., 2022). In combination with leukotriene receptor antagonists (e.g., montelukast sodium), it significantly reduces serum IgE levels and eosinophil ratios, especially in AR patients with asthma (Guo and Cao, 2023). In clinical practice, BUD is recommended as a first-line treatment for seasonal AR in children and adolescents, and its safety and efficacy have been confirmed in several studies (Ciprandi, 2024a; Li et al., 2024). The drug is not only effective in allergy-induced rhinitis but also significantly reduces the symptoms of nasal inflammation triggered by non-simple allergic factors such as air pollution, reflecting a broad anti-inflammatory mechanism of action. For patients with chronic rhinosinusitis combined with nasal polyps (CRSwNP) or asthma and other type 2 inflammation-related diseases (Guo and Cao, 2023; Ciprandi, 2024b), BUD can effectively improve nasal ventilation and control multidimensional symptoms by inhibiting pathological processes such as eosinophil infiltration.

In terms of improving QoL, CB and FLU have shown significant efficacy in AR patients compared to placebo (saline-based). In contemporary medical practice and healthcare, QoL, which is closely related to health, is considered a central concept (Seidl and Zannon, 2004). Nocturnal symptoms, in addition to the standard symptoms of nasal congestion, runny nose, and itching, are particularly common in patients with chronic diseases such as AR, which severely impacts their sleep (Juniper et al., 2003). Notably, sleep disturbance is a key area assessed in the RQLQ tool, which is our primary indicator of QoL. In the 1970s, a strong dependence relationship was reported between sleep patterns and good nasal function (Cottle, 1972). Nasal congestion is now considered a widely recognized etiologic factor for breathing disorders during sleep (Cottle, 1972). Therefore, by effectively addressing nighttime symptoms such as nasal congestion, sleep quality can be significantly improved, thereby meaningfully improving overall QoL (Brozek et al., 2010). Several surveys (Meltzer et al., 2009; Meltzer et al., 2012; Derebery et al., 2008) have shown that individuals with AR are more likely to be drowsy during the daytime due to poor sleep quality compared to non-AR patients, and are therefore unable to maintain sustained concentration, which in turn leads to reduced learning efficiency. About 30% of these cases have impaired cognitive and memory functions, and about 30% suffer from anxiety or depression. In terms of social work output, about 82% of adult patients had reduced work performance, while about 92% of pediatric patients had regressed in academic performance. In addition, fatigue, impaired sleep quality, inattention, and reduced productivity due to AR pose a huge financial stress on patients, covering medical expenses and additional costs triggered by sick leave. These consequences are exacerbated by poor symptom control or adverse drug reactions (Bernstein et al., 2024). Evidence suggests that improving QoL must be an important part of the treatment of AR. For AR patients, it is often difficult or impossible to completely avoid allergens. Therefore, most patients require continuous anti-inflammatory treatment to reduce symptoms (Wang et al., 2004). Compared to placebo, CB relieves all nasal symptoms and improves peak inspiratory flow, which can significantly improve patients’ QoL and alleviate their financial burden (Given et al., 2010; Nathan, 2007). Huangqi Jianzhong Tang (a famous Chinese herbal formula consisting of seven Chinese medicinal ingredients, including astragalus, cinnamon, licorice, white peony root, ginger, jujube, and Yitang) is commonly used to treat chronic inflammatory diseases such as chronic gastritis, inflammatory bowel disease, chronic hepatitis, and chronic nephritis, and has the same results as improving rhinitis (RQLQ score) (Zhang, 1995). Its high efficiency in addressing patients’ nasal symptoms may be related to the enrichment of plant-derived polyphenols (Hong et al., 2012).

Type-A proanthocynidins isolated from cinnamon bark have been shown to have therapeutic effects for immune inflammation, arthritis, asthma, and viral infections (Anderson et al., 2004; Joshi et al., 2000; Cao and Anderson, 2011). It has been reported that nasal administration of TAPP-CZ inhibits the recruitment of eosinophils infiltrating the nasal mucosa, which may be one of the anti-allergic mechanisms (Aswar et al., 2015). Another study further suggested that this may be related to its rich polyphenols inhibiting IgE-mediated mast cell degranulation (Kandhare et al., 2017). IL-4-dependent eosinophil recruitment leads to elevated inflammation in allergic diseases (Silvestri et al., 2006). A clinical study has shown that serum IL-4 levels are higher in patients with AR compared to healthy controls (Deo et al., 2010). These findings demonstrate that inhibiting the release of pro-inflammatory cytokines from mast cells is essential for relieving allergic inflammation, providing a reasonable pharmacological explanation for its ability to significantly improve patients’ QoL. Hydrogen-rich saline (HRS) primarily achieves its efficacy by increasing the expression level of Nrf2 protein and reducing the production of reactive oxygen species, thereby enhancing the body’s antioxidant stress response (Xie et al., 2014). The antioxidant and anti-inflammatory effects of hydrogen have been confirmed in vitro cell studies (Xu et al., 2018). It can reduce the levels of proinflammatory factors, thereby improving the barrier function and anti-inflammatory capacity of the nasal mucosa. Given this established mechanism, the high ranking of HRS (SUCRA = 82.58%, as presented in Figure 5) in our analysis suggests promising therapeutic potential. Despite this, its effect lacked statistical significance. We propose that the specific hydrogen concentration used might underlie this inconsistency, a hypothesis that must be addressed in subsequent studies. Fluticasone (FLU), a nasal corticosteroid, has potent anti-inflammatory effects and low absolute bioavailability (0.5%) (Allen et al., 2007). It activates intracellular glucocorticoid receptors to inhibit the transcription of multiple inflammatory genes, thereby comprehensively suppressing the activation of inflammatory cells and the release of inflammatory mediators. In summary, CB and FLU may collectively improve the QoL of patients with AR through different pathways, including regulating immune inflammation, combating oxidative stress, and exerting potent anti-inflammatory effects.

Previous studies have demonstrated that polyphenolic metabolites with therapeutic potential, such as RESV, inhibit the production of IgE antibodies (Kumazawa et al., 2014). Based on the data from the present study, in improving nasal symptoms, polyphenol-rich extracts may directly affect immune cells and modulate the pathogenesis of allergic inflammation through a unique mechanism of action (Enomoto et al., 2006). The effectiveness of this mechanism of action may be superior to that of nasal glucocorticosteroids (e.g., BUD), which are widely available on the market today. Therefore, an in-depth exploration of the underlying molecular mechanism of action is warranted.

The results of this study also provide insights into an important question in clinical practice: how to optimize treatment options for patients with severe symptoms who do not respond adequately to saline irrigation alone. Our network meta-analysis demonstrated that adding active ingredients such as resveratrol or cinnamon to irrigation solutions significantly relieved symptoms than placebo (saline). This suggests that medicated nasal irrigation could serve as an effective strategy for patients who require more intensive treatment. This, to some extent, shifts the traditional view (nasal irrigation has limited benefits for patients with severe disease) to the view that optimized nasal irrigation regimens may provide personalized treatment options for patients with varying degrees of disease severity. Future research should focus on identifying which patient subgroups would most benefit from these enhanced irrigation regimens.

The strength of this review lies in that this is the first systematic review of the most recent and comprehensive evidence on the use of different drugs for nasal irrigation. We also conducted a meta-analysis of high-quality RCTs.

There are some limitations to this review. First, most of the included studies did not describe specific randomization methods, allocation concealment, and blinded designs, which may reduce the reliability of the results to some extent. Second, due to the absence of closed loops between different drugs, we cannot explore in depth the sources of heterogeneity in comparisons of different interventions, which limits the interpretation of our results and needs to be confirmed by further studies. Third, we did not investigate the efficacy of combining multiple drugs in irrigation solutions (e.g., a budesonide and resveratrol compound solution). Examining the synergistic effects of such combinations would be a valuable future research direction. Fourth, multiple interventions (e.g., cinnamon) were reported in only one RCT. This increases the risk of overestimating the effect sizes of these specific interventions, and the results should be interpreted with caution. Further well-designed, original studies with sufficient sample sizes are needed to validate our findings.

## 5 Conclusion

This meta-analysis revealed that RESV was efficacious in reducing nasal symptoms, and CB was efficacious in improving QoL. These two relatively innovative traditional Chinese medicine treatments provide new ideas for the management of AR through multi-targeted modulation of immune homeostasis and anti-inflammatory effects, thus contributing to precision medicine and broadening the avenues for drug development. This may promote the acceptance and use of traditional Chinese medicine in clinical practice. Certainly, all interventions are more effective than placebo in managing symptoms in AR. In practice, physicians should choose treatment options based on the actual condition of the patient.

## Data Availability

The original contributions presented in the study are included in the article/Supplementary Material, further inquiries can be directed to the corresponding author.

## References

[B1] Allen A. Down G. Newland A. Reynard K. Rousell V. Salmon E. (2007). Absolute bioavailability of intranasal fluticasone furoate in healthy subjects. Clin. Ther. 29 (7), 1415–1420. 10.1016/j.clinthera.2007.07.028 17825692

[B2] Anderson R. A. Broadhurst C. L. Polansky M. M. Schmidt W. F. Khan A. Flanagan V. P. (2004). Isolation and characterization of polyphenol type-A polymers from cinnamon with insulin-like biological activity. J. Agric. Food Chem. 52 (1), 65–70. 10.1021/jf034916b 14709014

[B3] Aswar U. M. Kandhare A. D. Mohan V. Thakurdesai P. A. (2015). Anti-allergic effect of intranasal administration of type-A procyanidin polyphenols based standardized extract of cinnamon bark in ovalbumin sensitized BALB/c mice. Phytother. Res. 29 (3), 423–433. 10.1002/ptr.5269 25504814

[B4] Badorrek P. Hohlfeld J. M. Krug N. Joshi A. Raut A. (2015). Efficacy and safety of a novel nasal steroid, S0597, in patients with seasonal allergic rhinitis. Ann. Allergy Asthma Immunol. 115 (4), 325–329. 10.1016/j.anai.2015.07.016 26272281

[B5] Berkowitz R. B. Roberson S. Zora J. Capano D. Chen R. Lutz C. (1999). Mometasone furoate nasal spray is rapidly effective in the treatment of seasonal allergic rhinitis in an outdoor (park), acute exposure setting. Allergy Asthma Proc. 20 (3), 167–172. 10.2500/108854199778553037 10389549

[B6] Bernstein J. A. Bernstein J. S. Makol R. Ward S. (2024). Allergic rhinitis: a review. Jama 331 (10), 866–877. 10.1001/jama.2024.0530 38470381

[B7] Bousquet J. Neukirch F. Bousquet P. J. Gehano P. Klossek J. M. Le Gal M. (2006). Severity and impairment of allergic rhinitis in patients consulting in primary care. J. Allergy Clin. Immunol. 117 (1), 158–162. 10.1016/j.jaci.2005.09.047 16387600

[B8] Bousquet J. Khaltaev N. Cruz A. A. Denburg J. Fokkens W. J. Togias A. (2008). Allergic rhinitis and its impact on asthma (ARIA) 2008 update (in collaboration with the world health organization, GA(2)LEN and AllerGen). Allergy 63 (63 Suppl. 86), 8–160. 10.1111/j.1398-9995.2007.01620.x 18331513

[B9] Bozdemir K. Şahin E. Altintoprak N. Muluk N. B. Cengiz B. P. Acar M. (2016). Is resveratrol therapeutic when used to treat allergic rhinitisinitis in rats? Clin. Invest Med. 39 (2), E63–E72. 10.25011/cim.v39i2.26482 27040862

[B10] Brozek J. L. Bousquet J. Baena-Cagnani C. E. Bonini S. Canonica G. W. Casale T. B. (2010). Allergic rhinitis and its impact on asthma (ARIA) guidelines: 2010 revision. J. Allergy Clin. Immunol. 126 (3), 466–476. 10.1016/j.jaci.2010.06.047 20816182

[B11] Cai J. Tao P. Ding F. (2025). Enhanced the long-term impact of immunomodulatory treatment on the quality of life in patients suffering from allergic rhinitis. Med. Baltim. 104 (17), e42244. 10.1097/md.0000000000042244 40295274 PMC12039976

[B12] Cao H. Anderson R. A. (2011). Cinnamon polyphenol extract regulates tristetraprolin and related gene expression in mouse adipocytes. J. Agric. Food Chem. 59 (6), 2739–2744. 10.1021/jf103527x 21329350

[B13] Chen M. Fu Q. Song X. Muhammad A. Jia R. Zou Y. (2020). Preparation of resveratrol dry suspension and its immunomodulatory and anti-inflammatory activity in mice. Pharm. Biol. 58 (1), 8–15. 10.1080/13880209.2019.1699123 31847682 PMC6968662

[B14] Chong S. N. Chew F. T. (2018). Epidemiology of allergic rhinitis and associated risk factors in Asia. World Allergy Organ J. 11 (1), 17. 10.1186/s40413-018-0198-z 30128063 PMC6091170

[B15] Christodoulopoulos P. Cameron L. Durham S. Hamid Q. (2000). Molecular pathology of allergic disease. II: upper airway disease. J. Allergy Clin. Immunol. 105 (2 Pt 1), 211–223. 10.1016/s0091-6749(00)90068-x 10669839

[B16] Ciprandi G. (2024a). The updated role of budesonide in managing children and adolescents with allergic rhinitis. Minerva Pediatr. (Torino). 76 (4), 526–536. 10.23736/s2724-5276.24.07538-4 38407014

[B17] Ciprandi G. (2024b). Budesonide aqueous nasal spray: an updated review in managing chronic rhinosinusitis with nasal polyps. Panminerva Med. 66 (3), 317–323. 10.23736/s0031-0808.24.05200-5 39016712

[B18] Ciprandi G. Tosca M. A. Cirillo I. Vizzaccaro A. (2003). The effect of budesonide on the cytokine pattern in patients with perennial allergic rhinitis. Ann. Allergy Asthma Immunol. 91 (5), 467–471. 10.1016/s1081-1206(10)61515-3 14692430

[B19] Cottle M. H. (1972). Nasal breathing pressures and cardio-pulmonary illness. Eye Ear Nose Throat Mon. 51 (9), 331–340. 5068888

[B20] Coyte P. C. (1990). Current trends in Canadian health care: myths and misconceptions in health economics. J. Public Health Policy 11 (2), 169–188. 10.2307/3342750 2114422

[B21] Deo S. S. Mistry K. J. Kakade A. M. Niphadkar P. V. (2010). Role played by Th2 type cytokines in IgE mediated allergy and asthma. Lung India 27 (2), 66–71. 10.4103/0970-2113.63609 20616938 PMC2893428

[B22] Derebery J. Meltzer E. Nathan R. A. Stang P. E. Campbell U. B. Corrao M. (2008). Rhinitis symptoms and comorbidities in the United States: burden of rhinitis in America survey. Otolaryngol. Head. Neck Surg. 139 (2), 198–205. 10.1016/j.otohns.2008.05.019 18656715

[B23] Dykewicz M. S. Kaiser H. B. Nathan R. A. Goode-Sellers S. Cook C. K. Witham L. A. (2003). Fluticasone propionate aqueous nasal spray improves nasal symptoms of seasonal allergic rhinitis when used as needed (prn). Ann. Allergy Asthma Immunol. 91 (1), 44–48. 10.1016/s1081-1206(10)62057-1 12877448

[B24] Ellis A. K. Steacy L. M. Joshi A. Bhowmik S. Raut A. (2016). Efficacy of the novel nasal steroid S0597 tested in an environmental exposure unit. Ann. Allergy Asthma Immunol. 117 (3), 310–317. 10.1016/j.anai.2016.07.018 27613466

[B25] Enomoto T. Nagasako-Akazome Y. Kanda T. Ikeda M. Dake Y. (2006). Clinical effects of Apple polyphenols on persistent allergic rhinitis: a randomized double-blind placebo-controlled parallel arm study. J. Investig. Allergol. Clin. Immunol. 16 (5), 283–289. 17039666

[B26] Gendeh H. S. Hamizan A. W. Husain S. Nawi A. M. Zahedi F. D. Megat Ismail N. F. (2024). The efficacy of elonide nasal corticosteroids in managing allergic rhinitis: a randomized, double-blinded trial. J. Clin. Med. 13 (7), 1883. 10.3390/jcm13071883 38610648 PMC11012514

[B27] Given J. T. Cheema A. S. Dreykluft T. Stillerman A. Silvey M. Wu W. (2010). Fluticasone furoate nasal spray is effective and well tolerated for perennial allergic rhinitis in adolescents and adults. Am. J. Rhinol. Allergy 24 (6), 444–450. 10.2500/ajra.2010.24.3534 21144223

[B28] Guo S. Cao C. (2023). Effects of montelukast sodium combined with budesonide on pulmonary function, serum IgE levels, and EOS percentage in children with comorbid allergic rhinitis and asthma. Am. J. Transl. Res. 15 (12), 6823–6831. 38186993 PMC10767537

[B29] Ha J. Lee S. W. Yon D. K. (2020). Ten-year trends and prevalence of asthma, allergic rhinitis, and atopic dermatitis among the Korean population, 2008-2017. Clin. Exp. Pediatr. 63 (7), 278–283. 10.3345/cep.2019.01291 32023407 PMC7374008

[B30] Hampel F. C. Jr. Ratner P. H. Amar N. J. van Bavel J. H. Mohar D. Fairchild C. J. (2006). Improved quality of life among seasonal allergic rhinitis patients treated with olopatadine HCl nasal spray 0.4% and olopatadine HCl nasal spray 0.6% compared with vehicle placebo. Allergy Asthma Proc. 27 (3), 202–207. 10.2500/aap.2006.27.2862 16913262

[B31] Han S. Y. Choi Y. J. Kang M. K. Park J. H. Kang Y. H. (2015). Resveratrol suppresses cytokine production linked to FcεRI-MAPK activation in IgE-Antigen complex-exposed basophilic mast cells and mice. Am. J. Chin. Med. 43 (8), 1605–1623. 10.1142/s0192415x15500913 26621445

[B32] Hermelingmeier K. E. Weber R. K. Hellmich M. Heubach C. P. Mösges R. (2012). Nasal irrigation as an adjunctive treatment in allergic rhinitis: a systematic review and meta-analysis. Am. J. Rhinol. Allergy 26 (5), e119–e125. 10.2500/ajra.2012.26.3787 23168142 PMC3904042

[B33] Hong J. W. Yang G. E. Kim Y. B. Eom S. H. Lew J. H. Kang H. (2012). Anti-inflammatory activity of cinnamon water extract *in vivo* and *in vitro* LPS-induced models. BMC Complement. Altern. Med. 12, 237. 10.1186/1472-6882-12-237 23190501 PMC3533872

[B34] Hozo S. P. Djulbegovic B. Hozo I. (2005). Estimating the mean and variance from the median, range, and the size of a sample. BMC Med. Res. Methodol. 5, 13. 10.1186/1471-2288-5-13 15840177 PMC1097734

[B35] Hu C. Li L. (2019). The application of resveratrol to mesenchymal stromal cell-based regenerative medicine. Stem Cell Res. Ther. 10 (1), 307. 10.1186/s13287-019-1412-9 31623691 PMC6798337

[B36] Hutton B. Salanti G. Caldwell D. M. Chaimani A. Schmid C. H. Cameron C. (2015). The PRISMA extension statement for reporting of systematic reviews incorporating network meta-analyses of health care interventions: checklist and explanations. Ann. Intern Med. 162 (11), 777–784. 10.7326/m14-2385 26030634

[B37] Jahan H. Yong J. (2018). Effect of 38°C hypertonic saline nasal irrigation on the efficacy and prognosis of glucocorticoid therapy. Chongqing Med. 47 (30), 3909–3912. 10.3969/j.issn.1671-8348.2018.30.016

[B38] Jin L. Yu S. Q. Zhang X. Ge Q. Zhang X. L. Wang Y. (2018). Clinical study of hydrogen-rich saline in the treatment of moderate to severe allergic rhinitis. J. Clin. Otorhinolaryngology Head Neck Surg. 32 (7), 493–496. 10.13201/j.issn.1001-1781.2018.07.004 29798076

[B39] Jin L. Fan K. Tan S. Liu S. Ge Q. Wang Y. (2022). The beneficial effects of hydrogen-rich saline irrigation on chronic rhinitis: a randomized, double-blind clinical trial. J. Inflamm. Res. 15, 3983–3995. 10.2147/jir.S365611 35873384 PMC9296884

[B40] Joshi S. S. Kuszynski C. A. Bagchi M. Bagchi D. (2000). Chemopreventive effects of grape seed proanthocyanidin extract on chang liver cells. Toxicology 155 (1-3), 83–90. 10.1016/s0300-483x(00)00280-8 11154800

[B41] Juniper E. F. Rohrbaugh T. Meltzer E. O. (2003). A questionnaire to measure quality of life in adults with nocturnal allergic rhinoconjunctivitis. J. Allergy Clin. Immunol. 111 (3), 484–490. 10.1067/mai.2003.137 12642826

[B42] Kandhare A. D. Aswar U. M. Mohan V. Thakurdesai P. A. (2017). Ameliorative effects of type-A procyanidins polyphenols from cinnamon bark in compound 48/80-induced mast cell degranulation. Anat. Cell Biol. 50 (4), 275–283. 10.5115/acb.2017.50.4.275 29354299 PMC5768564

[B43] Kanjanawasee D. Seresirikachorn K. Chitsuthipakorn W. Snidvongs K. (2018). Hypertonic saline Versus isotonic saline nasal irrigation: systematic review and meta-analysis. Am. J. Rhinol. Allergy. 32 (4), 269–279. 10.1177/1945892418773566 29774747

[B44] Kim H. C. Kim D. K. Kim J. S. Lee H. J. Bae M. R. Choi W. R. (2022). Hypochlorous acid Versus saline nasal irrigation in allergic rhinitis: a multicenter, randomized, double-blind, placebo-controlled study. Am. J. Rhinol. Allergy 36 (1), 129–134. 10.1177/19458924211029428 34236253

[B45] Kumazawa Y. Takimoto H. Matsumoto T. Kawaguchi K. (2014). Potential use of dietary natural products, especially polyphenols, for improving type-1 allergic symptoms. Curr. Pharm. Des. 20 (6), 857–863. 10.2174/138161282006140220120344 23701564

[B46] Li Y. Xiong J. Zhang Z. Liao K. Zho X. Li J. (2024). Efficacy and safety of various corticosteroids in the treatment of children with allergic rhinitis: a systematic review and network meta-analysis. J. Evid. Based Med. 17 (3), 626–642. 10.1111/jebm.12645 39313999

[B47] London N. R. Jr. Ramanathan M. Jr (2017). The role of the sinonasal epithelium in allergic rhinitis. Otolaryngol. Clin. North Am. 50 (6), 1043–1050. 10.1016/j.otc.2017.08.002 28988814 PMC5752131

[B48] Long D. Q. (2013). Study on the efficacy of compound eqi nasal irrigation fluid in treating allergic rhinitis. China Med. Pharm. 3 (12), 195–196.

[B49] Luo X. Hua Z. X. Zhang Y. N. Yang Q. T. (2024). Review of the development and latest perspectives in 2024 on allergic rhinitis and its impact on asthma (ARIA). Zhonghua Er Bi Yan Hou Tou Jing Wai Ke Za Zhi 59 (10), 1107–1114. 10.3760/cma.j.cn115330-20240725-00450 39472126

[B50] Lv C. Zhang Y. Shen L. (2018). Preliminary clinical effect evaluation of resveratrol in adults with allergic rhinitis. Int. Arch. Allergy Immunol. 175 (4), 231–236. 10.1159/000486959 29539616

[B51] Magen E. Yosefy C. Viskoper R. J. Mishal J. (2006). Treatment of allergic rhinitis can improve blood pressure control. J. Hum. Hypertens. 20 (11), 888–893. 10.1038/sj.jhh.1002088 16967045

[B52] Meltzer E. O. Blaiss M. S. Derebery M. J. Mahr T. A. Gordon B. R. Sheth K. K. (2009). Burden of allergic rhinitis: results from the pediatric allergies in America survey. J. Allergy Clin. Immunol. 124 (3 Suppl. l), S43–S70. 10.1016/j.jaci.2009.05.013 19592081

[B53] Meltzer E. O. Blaiss M. S. Naclerio R. M. Stoloff S. W. Derebery M. J. Nelson H. S. (2012). Burden of allergic rhinitis: allergies in America, Latin America, and Asia-Pacific adult surveys. Allergy Asthma Proc. 33 (Suppl. 1), S113–S141. 10.2500/aap.2012.33.3603 22981425

[B54] Min Y. G. (2010). The pathophysiology, diagnosis and treatment of allergic rhinitis. Allergy Asthma Immunol. Res. 2 (2), 65–76. 10.4168/aair.2010.2.2.65 20358020 PMC2846743

[B55] Mullol J. Obando A. Pujols L. Alobid I. (2009). Corticosteroid treatment in chronic rhinosinusitis: the possibilities and the limits. Immunol. Allergy Clin. North Am. 29 (4), 657–668. 10.1016/j.iac.2009.07.001 19879441

[B56] Munch E. P. Søborg M. Nørreslet T. T. Mygind N. (1983). A comparative study of dexchlorpheniramine maleate sustained release tablets and budesonide nasal spray in seasonal allergic rhinitis. Allergy 38 (7), 517–524. 10.1111/j.1398-9995.1983.tb02361.x 6139040

[B57] Naclerio R. M. (1991). Allergic rhinitis. N. Engl. J. Med. 325 (12), 860–869. 10.1056/nejm199109193251206 1875971

[B58] Nathan R. A. (2007). The burden of allergic rhinitis. Allergy Asthma Proc. 28 (1), 3–9. 10.2500/aap.2007.28.2934 17390749

[B59] Nielsen L. P. Mygind N. Dahl R. (2001). Intranasal corticosteroids for allergic rhinitis: superior relief? Drugs 61 (11), 1563–1579. 10.2165/00003495-200161110-00004 11577794

[B60] Patel G. B. Kern R. C. Bernstein J. A. Hae-Sim P. Peters A. T. (2020). Current and future treatments of rhinitis and sinusitis. J. Allergy Clin. Immunol. Pract. 8 (5), 1522–1531. 10.1016/j.jaip.2020.01.031 32004747 PMC7416524

[B61] Pawankar R. Yamagishi S. Yagi T. (2000). Revisiting the roles of mast cells in allergic rhinitis and its relation to local IgE synthesis. Am. J. Rhinol. 14 (5), 309–317. 10.2500/105065800781329582 11068656

[B62] Pawankar R. Mori S. Ozu C. Kimura S. (2011). Overview on the pathomechanisms of allergic rhinitis. Asia Pac Allergy 1 (3), 157–167. 10.5415/apallergy.2011.1.3.157 22053313 PMC3206239

[B63] Ratner P. H. Wingertzahn M. A. van Bavel J. H. Hampel F. Darken P. F. Shah T. (2006). Efficacy and safety of ciclesonide nasal spray for the treatment of seasonal allergic rhinitis. J. Allergy Clin. Immunol. 118 (5), 1142–1148. 10.1016/j.jaci.2006.07.050 17088141

[B64] Rimmer J. Greenwood A. Bartlett D. Hellgren J. (2012). Nasal steroids improve regulation of nasal patency in asthma and mild rhinitis: a randomised, cross-over trial. Eur. Arch. Otorhinolaryngol. 269 (4), 1133–1138. 10.1007/s00405-011-1803-8 22033572

[B65] Rinne J. Simola M. Malmberg H. Haahtela T. (2002). Early treatment of perennial rhinitis with budesonide or cetirizine and its effect on long-term outcome. J. Allergy Clin. Immunol. 109 (3), 426–432. 10.1067/mai.2002.121703 11897986

[B66] Sansila K. Eiamprapai P. Sawangjit R. (2020). Effects of self-prepared hypertonic nasal saline irrigation in allergic rhinitis: a randomized controlled trial. Asian Pac J. Allergy Immunol. 38 (3), 200–207. 10.12932/ap-090618-0331 30525740

[B67] Seidl E. M. Zannon C. M. (2004). Quality of life and health: conceptual and methodological issues. Cad. Saude Publica 20 (2), 580–588. 10.1590/s0102-311x2004000200027 15073639

[B68] Shen C. Y. Jiang J. G. Yang L. Wang D. W. Zhu W. (2017). Anti-ageing active ingredients from herbs and nutraceuticals used in traditional Chinese medicine: pharmacological mechanisms and implications for drug discovery. Br. J. Pharmacol. 174 (11), 1395–1425. 10.1111/bph.13631 27659301 PMC5429334

[B69] Silvestri M. Bontempelli M. Giacomelli M. Malerba M. Rossi G. A. Di Stefano A. (2006). High serum levels of tumour necrosis factor-alpha and interleukin-8 in severe asthma: markers of systemic inflammation? Clin. Exp. Allergy 36 (11), 1373–1381. 10.1111/j.1365-2222.2006.02502.x 17083347

[B70] Singh R. Galagali J. Kumar S. Bahurupi Y. Chandrachood M. (2016). Comparative study of intranasal hypertonic seawater saline versus intranasal normal saline in allergic rhinitis. Int. J. Otorhinolaryngology Head Neck Surg. 3, 104. 10.18203/issn.2454-5929.ijohns20164810

[B71] Small P. Keith P. K. Kim H. (2018). Allergic rhinitis. Allergy Asthma Clin. Immunol. 14 (Suppl. 2), 51. 10.1186/s13223-018-0280-7 30263033 PMC6156899

[B72] Snidvongs K. Thanaviratananich S. (2017). Update on intranasal medications in rhinosinusitis. Curr. Allergy Asthma Rep. 17 (7), 47. 10.1007/s11882-017-0720-3 28602009

[B73] Steels E. Steels E. Deshpande P. Thakurdesai P. Dighe S. Collet T. (2019). A randomized, double-blind placebo-controlled study of intranasal standardized cinnamon bark extract for seasonal allergic rhinitis. Complementary Ther. Med. 47, 102198. 10.1016/j.ctim.2019.102198 31780001

[B74] Toppila-Salmi S. van Drunen C. M. Fokkens W. J. Golebski K. Mattila P. Joenvaara S. (2015). Molecular mechanisms of nasal epithelium in rhinitis and rhinosinusitis. Curr. Allergy Asthma Rep. 15 (2), 495. 10.1007/s11882-014-0495-8 25504259 PMC4262789

[B75] Wang L. L. (2016). Efficacy of Xanthium sibiricum wash on Lung-Qi deficiency type allergic rhinitis and its impact on T-Lymphocyte subsets. J. Bethune Med. Sci. 14 (4), 471–472. 10.16485/j.issn.2095-7858.2016.04.031

[B76] Wang D. Y. Raza M. T. Gordon B. R. (2004). Control of nasal obstruction in perennial allergic rhinitis. Curr. Opin. Allergy Clin. Immunol. 4 (3), 165–170. 10.1097/00130832-200406000-00005 15126936

[B77] Wheatley L. M. Togias A. (2015). Clinical practice. Allergic rhinitis. N. Engl. J. Med. 372 (5), 456–463. 10.1056/NEJMcp1412282 25629743 PMC4324099

[B78] Wise S. K. Lin S. Y. Toskala E. Orlandi R. R. Akdis C. A. Alt J. A. (2018). International consensus statement on allergy and rhinology: allergic rhinitis. Int. Forum Allergy Rhinol. 8 (2), 108–352. 10.1002/alr.22073 29438602 PMC7286723

[B79] Wu F. H. Zhu D. H. Liu G. (2012). Clinical observation of compound eqi nasal irrigation fluid in treating allergic rhinitis and its effect on nasal mucociliary clearance time. J. Extern. Ther. Traditional Chin. Med. 21 (5), 8–10. 10.3969/j.issn.1006-978X.2012.05.004

[B80] Wu M. Wang Q. Zhang K. Wu K. Zhang Y. Wang Z. (2014). The effect of nasal irrigation in the treatment of allergic rhinitis. Lin. Chuang Er Bi Yan Hou Tou Jing Wai Ke Za Zhi 28 (5), 287–289. 25185277

[B81] Xaubet A. Mullol J. Roca-Ferrer J. Pujols L. Fuentes M. Pérez M. (2001). Effect of budesonide and nedocromil sodium on IL-6 and IL-8 release from human nasal mucosa and polyp epithelial cells. Respir. Med. 95 (5), 408–414. 10.1053/rmed.2001.1061 11392584

[B82] Xie W. Du L. (2011). Diabetes is an inflammatory disease: evidence from traditional Chinese medicines. Diabetes Obes. Metab. 13 (4), 289–301. 10.1111/j.1463-1326.2010.01336.x 21205111

[B83] Xie Q. Li X. X. Zhang P. Li J. C. Cheng Y. Feng Y. L. (2014). Hydrogen gas protects against serum and glucose deprivation-induced myocardial injury in H9c2 cells through activation of the NF-E2-related factor 2/heme oxygenase 1 signaling pathway. Mol. Med. Rep. 10 (2), 1143–1149. 10.3892/mmr.2014.2283 24890947

[B84] Xing C. Wang Y. Dai X. Yang F. Luo J. Liu P. (2020). The protective effects of resveratrol on antioxidant function and the mRNA expression of inflammatory cytokines in the ovaries of hens with fatty liver hemorrhagic syndrome. Poult. Sci. 99 (2), 1019–1027. 10.1016/j.psj.2019.10.009 32036959 PMC7587695

[B85] Xu F. Yu S. Qin M. Mao Y. Jin L. Che N. (2018). Hydrogen-rich saline ameliorates allergic rhinitis by reversing the imbalance of Th1/Th2 and Up-Regulation of CD4+CD25+Foxp3+Regulatory T cells, Interleukin-10, and membrane-bound transforming growth Factor-β in Guinea pigs. Inflammation 41 (1), 81–92. 10.1007/s10753-017-0666-6 28894978

[B86] Xu Y. Liu Q. Guo X. Xiang L. Zhao G. (2020). Resveratrol attenuates IL-33-induced mast cell inflammation associated with inhibition of NF-κB activation and the P38 signaling pathway. Mol. Med. Rep. 21 (3), 1658–1666. 10.3892/mmr.2020.10952 32016471

[B87] Yamada T. Yamamoto H. Kubo S. Sakashita M. Tokunaga T. Susuki D. (2012). Efficacy of mometasone furoate nasal spray for nasal symptoms, quality of life, rhinitis-disturbed sleep, and nasal nitric oxide in patients with perennial allergic rhinitis. Allergy Asthma Proc. 33 (2), e9–e16. 10.2500/aap.2012.33.3509 22525384

[B88] Yorgancıoğlu A. Özdemir C. Kalaycı Ö. Kalyoncu A. F. Bachert C. Baena-Cagnani C. E. (2012). ARIA (allergic rhinitis and its impact on asthma) achievements in 10 years and future needs. Tuberk. Toraks 60 (1), 92–97. 10.5578/tt.3734 22554377

[B89] Zhang Z. (1995). Synopsis of prescriptions of the golden chamber with 300 cases. Beijing, China: New World Press.

[B90] Zhang M. X. (2023). Therapeutic efficacy of hydrogen-rich saline nasal irrigation on allergic rhinitis and its impact on nasal exhaled nitric oxide levels. Contemp. Med. Symp. 21 (8), 118–121. 10.3969/j.issn.2095-7629.2023.08.035

[B91] Zhang D. X. Yao X. (2013). Treatment of 60 cases of allergic rhinitis with biqingling nasal irrigation. J. Shandong Univ. Traditional Chin. Med. 37 (02), 132–133. 10.16294/j.cnki.1007-659x.2013.02.018

[B92] Zhang J. Cheng H. Luo Y. Kan D. Wang Y. (2022). Effect of loratadine tablets in combination with other drugs on nasal physiological function and T lymphocyte subsets in patients with allergic rhinitis. Comput. Intell. Neurosci. 2022, 3990427. 10.1155/2022/3990427 36045965 PMC9420603

