# Supplementary material for: Efficacy of different nasal irrigation treatments versus placebo in allergic rhinitis: a systematic review and network meta-analysis
Source: Front Pharmacol. 2025 Nov 10;16:1670372. doi: 10.3389/fphar.2025.1670372 (PMC12641607; doi:10.3389/fphar.2025.1670372)
Supplement: Supplementary file 1 [file Supplementaryfile1.docx]

Supplementary Material

# Supplementary Figures and Tables

## Supplementary Figures


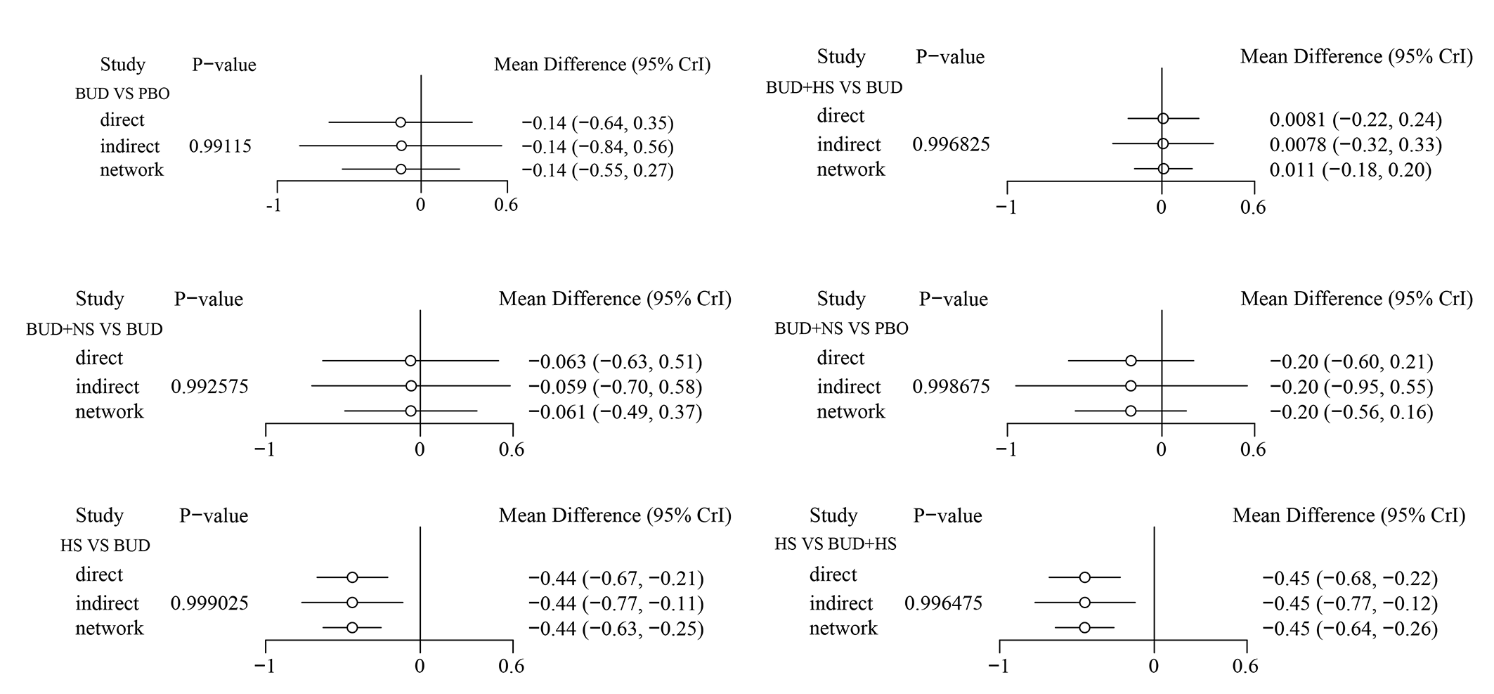


**Supplementary Figure 1.** RQLQ inconsistency test


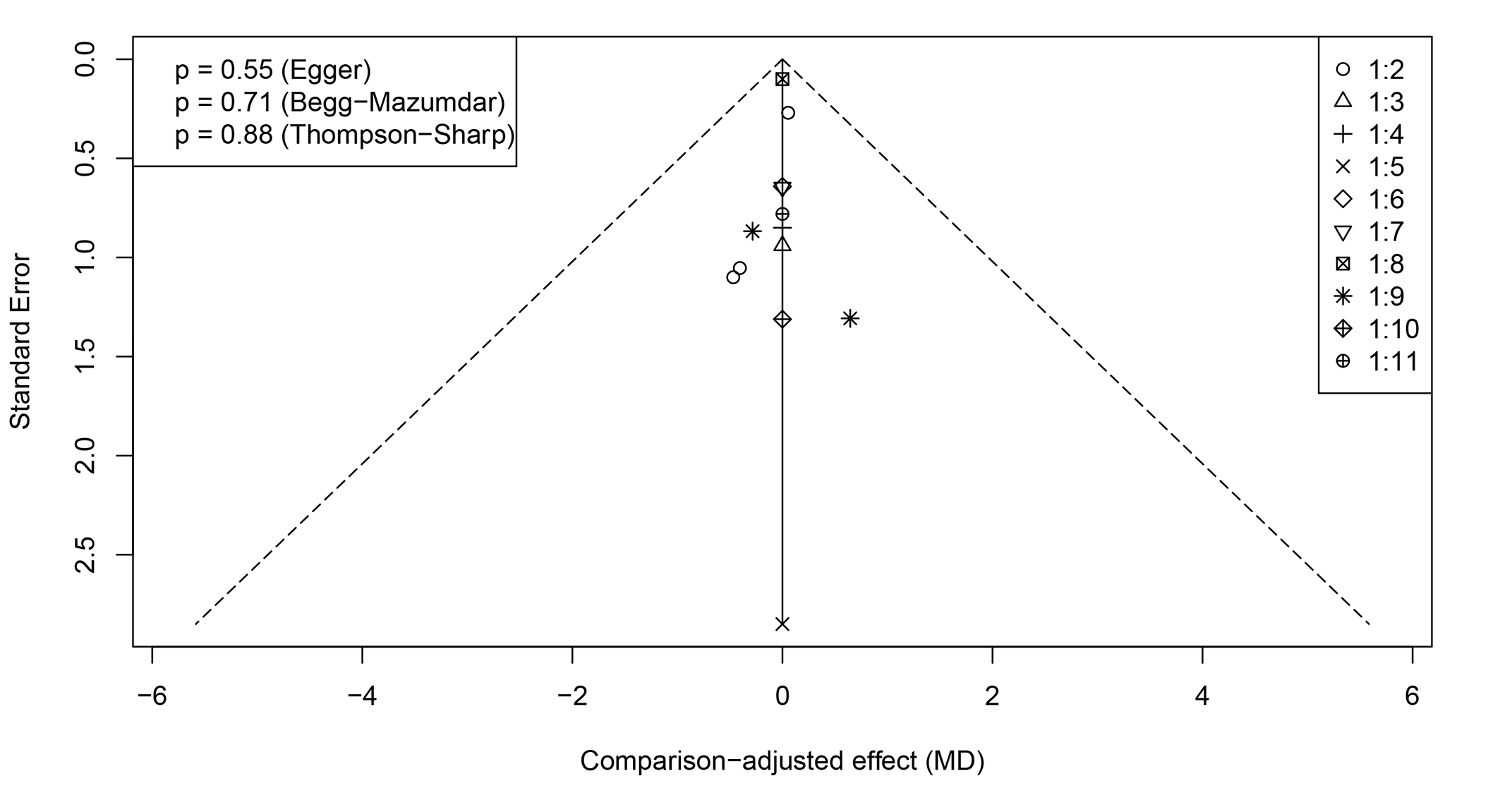


**Supplementary Figure 2.** Funnel plot of the RQLQ


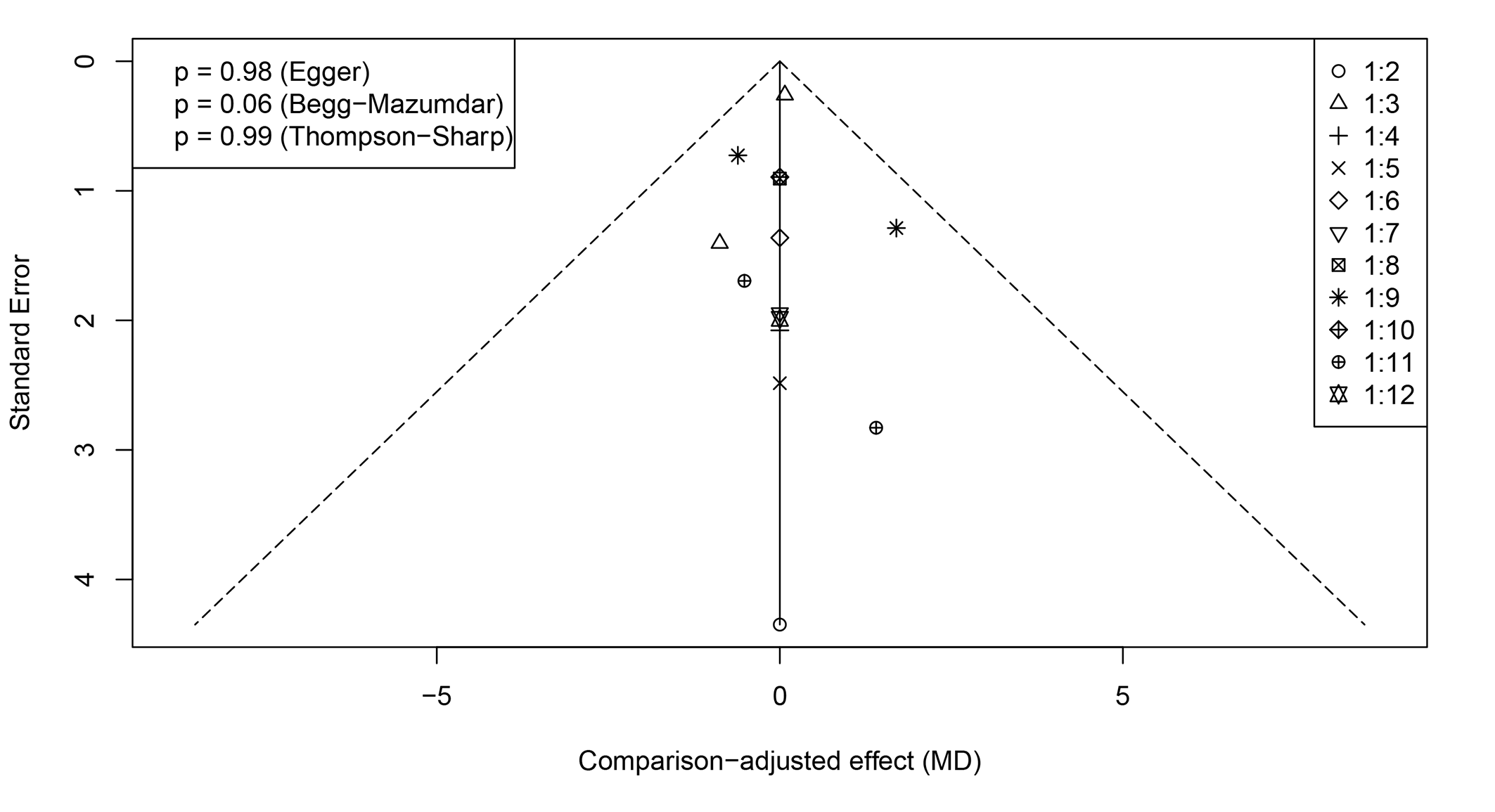


**Supplementary Figure 3.** Funnel plot of the TNSS

## Supplementary Tables

Supplementary Table 1. Search strategy

| Pubmed | | |
| --- | --- | --- |
| # | Query | Results |
| 1 | "Rhinitis, Allergic"[Mesh] | 24800 |
| 2 | Allergic Rhinitides[Title/Abstract] OR Allergic Rhinitis[Title/Abstract] OR allergic rhinopathy[Title/Abstract] OR atopic rhinitis[Title/Abstract] OR eosinophil rhinitis[Title/Abstract] OR eosinophile rhinitis[Title/Abstract] OR eosinophilic rhinitis[Title/Abstract] OR eosinophilous rhinitis[Title/Abstract] OR rhinitis allergica[Title/Abstract] OR rhinitis atopica[Title/Abstract] OR rhinitis eosinophila[Title/Abstract] | 24152 |
| 3 | Rinse[Title/Abstract] OR Irriga*[Title/Abstract] OR Lavage[Title/Abstract] OR Flush[Title/Abstract] OR spray[Title/Abstract] OR washing[Title/Abstract] | 193384 |
| 4 | nasal[Title/Abstract] | 150191 |
| 5 | (nasal[Title/Abstract]) AND (Rinse[Title/Abstract] OR Irriga*[Title/Abstract] OR Lavage[Title/Abstract] OR Flush[Title/Abstract] OR spray[Title/Abstract] OR washing[Title/Abstract]) | 8822 |
| 6 | ("Rhinitis, Allergic"[Mesh]) OR (Allergic Rhinitides[Title/Abstract] OR Allergic Rhinitis[Title/Abstract] OR allergic rhinopathy[Title/Abstract] OR atopic rhinitis[Title/Abstract] OR eosinophil rhinitis[Title/Abstract] OR eosinophile rhinitis[Title/Abstract] OR eosinophilic rhinitis[Title/Abstract] OR eosinophilous rhinitis[Title/Abstract] OR rhinitis allergica[Title/Abstract] OR rhinitis atopica[Title/Abstract] OR rhinitis eosinophila[Title/Abstract]) | 35295 |
| 7 | (("Rhinitis, Allergic"[Mesh]) OR (Allergic Rhinitides[Title/Abstract] OR Allergic Rhinitis[Title/Abstract] OR allergic rhinopathy[Title/Abstract] OR atopic rhinitis[Title/Abstract] OR eosinophil rhinitis[Title/Abstract] OR eosinophile rhinitis[Title/Abstract] OR eosinophilic rhinitis[Title/Abstract] OR eosinophilous rhinitis[Title/Abstract] OR rhinitis allergica[Title/Abstract] OR rhinitis atopica[Title/Abstract] OR rhinitis eosinophila[Title/Abstract])) AND ((nasal[Title/Abstract]) AND (Rinse[Title/Abstract] OR Irriga*[Title/Abstract] OR Lavage[Title/Abstract] OR Flush[Title/Abstract] OR spray[Title/Abstract] OR washing[Title/Abstract])) | 1962 |

| Embase | | |
| --- | --- | --- |
| # | Query | Results |
| 1 | 'allergic rhinitis'/exp | 62695 |
| 2 | 'allergic rhinitides':ab,ti OR 'allergic rhinitis':ab,ti OR 'allergic rhinopathy':ab,ti OR 'atopic rhinitis':ab,ti OR 'eosinophil rhinitis':ab,ti OR 'eosinophile rhinitis':ab,ti OR 'eosinophilic rhinitis':ab,ti OR 'eosinophilous rhinitis':ab,ti OR 'rhinitis allergica':ab,ti OR 'rhinitis atopica':ab,ti OR 'rhinitis eosinophila':ab,ti | 35160 |
| 3 | #1 OR #2 | 67137 |
| 4 | rinse:ab,ti OR irriga*:ab,ti OR lavage:ab,ti OR flush:ab,ti OR spary:ab,ti OR washing:ab,ti | 207403 |
| 5 | nasal:ab,ti | 199186 |
| 6 | #4 AND #5 | 6418 |
| 7 | #3 AND #6 | 1265 |

| Cochrane Library | | |
| --- | --- | --- |
| # | Query | Results |
| 1 | MeSH descriptor: [Rhinitis, Allergic] explode all trees | 3748 |
| 2 | (Allergic Rhinitides OR Allergic Rhinitis OR allergic rhinopathy OR atopic rhinitis OR eosinophil rhinitis OR eosinophile rhinitis OR eosinophilic rhinitis OR eosinophilous rhinitis OR rhinitis allergica OR rhinitis atopica OR rhinitis eosinophila):ti,ab,kw | 9230 |
| 3 | #1 or #2 | 9232 |
| 4 | (Rinse OR Irriga* OR Lavage OR Flush OR spary OR washing):ti,ab,kw | 19950 |
| 5 | (nasal):ti,ab,kw | 25472 |
| 6 | #4 AND #5 | 1392 |
| 7 | #3 AND #6 | 392 |

| Web of science | | |
| --- | --- | --- |
| # | Query | Results |
| 1 | TS=(Rhinitis, Allergic) | 31712 |
| 2 | "TS=(Allergic Rhinitides OR Allergic Rhinitis OR allergic rhinopathy OR atopic rhinitis OR eosinophil rhinitis OR eosinophile rhinitis OR eosinophilic rhinitis OR eosinophilous rhinitis OR rhinitis allergica OR rhinitis atopica OR rhinitis eosinophila ) " | 32694 |
| 3 | #2 OR #1 | 32694 |
| 4 | TS=(Rinse OR Irriga* OR Lavage OR Flush OR spary OR washing) | 454836 |
| 5 | TS=(nasal) | 157873 |
| 6 | #5 AND #4 | 7047 |
| 7 | #6 AND #3 | 1304 |

| CBM | | |
| --- | --- | --- |
| # | Query | Results |
| 1 | "Nasal"[Abstract: Intelligence] | 27697 |
| 2 | "Flushing "[Abstract: Intelligent] | 61583 |
| 3 | "Rhinitis, allergic "[unweighted: Extension] | 17013 |
| 4 | (#3) AND (#2) AND (#1) | 304 |

| VIP Database | |
| --- | --- |
| Query | Results |
| Abstract = allergic rhinitis + allergic rhinitis + perennial allergic rhinitis + perennial allergic rhinitis + perennial allergic rhinitis + perennial allergic rhinitis + perennial allergic rhinitis + perennial allergic rhinitis + allergic rhinitis + allergic rhinitis AND abstract = nasal irrigation | 213 |

| CNKI | |
| --- | --- |
| Query | Results |
| (Article summary: Allergic rhinitis (exact)) AND (Article summary: nasal cavity (exact)) AND (Article summary: irrigation (exact)) | 199 |

**Supplementary Table 2.** Detailed Description and Taxonomic Validation of Herbal and Natural Product Interventions

| Reference | Intervention | Latin binomial with authority [Family; Pharmacopoeial drugname] | Type of extract (if reported) | Source |
| --- | --- | --- | --- | --- |
| Chunjiang Lv 2018 | Resveratrol（Extracted from Polygonum cuspidatum） | *Reynoutria japonica* Houtt. [Polygonaceae; Polygoni cuspidati rhizoma et radix] | No reported | Kew Science |
| Eleanor Steels 2019 | Cinnamon Bark | *Cinnamomum verum* J.Presl [Lauraceae; Cinnamomi cortex] | Standardized to total polyphenols not less than 40 µg per 100 µL spray | Kew Science |
| Dongxia Zhang 2013 | Nose Clearing | *Houttuynia cordata* Thunb. [Saururaceae; Houttuyniae Herba] | Aqueous extract | Kew Science |
|  |  | *Scutellaria baicalensis* Georgi [Lamiaceae; Scutellariae radix] |  | Kew Science |
|  |  | *Nepeta tenuifolia* Benth. [Lamiaceae; Schizonepetae herba carbonisata] |  | Kew Science |
|  |  | *Xanthium strumarium* L. [Asteraceae; Xanthii fructus] |  | Kew Science |
|  |  | *Conioselinum anthriscoides 'Chuanxiong'* [Apiaceae; Chuanxiong rhizoma] |  | Kew Science |
|  |  | *Poria cocos* F.A.Wolf |  | GBIF |
|  |  | *Acorus verus* (L.) Raf. [Acoraceae; Acori tatarinowii rhizoma] |  | Kew Science |
| Dongqiu Long 2013/Feihu Wu 2012 | E-Qi | *Nepeta tenuifolia* Benth. [Lamiaceae; Schizonepetae herba carbonisata] | Water decoction (aqueous extract) | Kew Science |
|  |  | *Saposhnikovia divaricata* (Turcz. ex Ledeb.) Schischk. [Apiaceae; Saposhnikoviae radix] |  | Kew Science |
|  |  | *Magnolia biondii* Pamp. [Magnoliaceae; Magnoliae flos] |  | Kew Science |
|  |  | *Mentha canadensis* L. [Lamiaceae; Menthae Haplocalycis Herba] |  | Kew Science |
|  |  | *Angelica dahurica* (Hoffm.) Benth. & Hook.f. ex Franch. & Sav. [Apiaceae; Angelicae Dahuricae Radix] |  | Kew Science |
|  |  | *Vincetoxicum mukdenense* Kitag. [Apocynaceae; Cynanchi paniculati radix et rhizoma] |  | Kew Science |
|  |  | *Centipeda minima* (L.) A.Braun & Asch. [Asteraceae; Centipedae herba] |  | Kew Science |
|  |  | *Astragalus mongholicus* Bunge [Fabaceae; Astragali radix] |  | Kew Science |
|  |  | *Xanthium strumarium* L. [Asteraceae; Xanthii fructus] |  | Kew Science |
|  |  | *Punica granatum* L. [Lythraceae; Granati Pericarpium] |  | Kew Science |
| Lili Wang 2016 | Xanthium | *Xanthium strumarium* L. [Asteraceae; Xanthii fructus] | Aqueous extract | Kew Science |

GBIF: Global Biodiversity Information Facility

**Supplementary Table 3.** The results of RQLQ sensitivity analysis

| Fixed |  | Random |  | Eliminate low quality score | SUCRA |
| --- | --- | --- | --- | --- | --- |
| Treatment | SUCRA | Treatment | SUCRA | Treatment | SUCRA |
| PBO | 0.06728875 | PBO | 0.1370087 | PBO | 0.0707525 |
| FLU | **0.78551375** | FLU | **0.6985162** | FLU | **0.8152512** |
| BUD | 0.20047125 | BUD | 0.2602687 | BUD | 0.2086750 |
| BUD+NS | 0.25550250 | BUD+NS | 0.3128525 | BUD+NS | 0.2666975 |
| HRS | **0.90395250** | HRS | **0.8627050** | HRS | **0.8263150** |
| BUD+HS | 0.19252750 | BUD+HS | 0.2641375 | BUD+HS | 0.1980550 |
| HS | 0.63021625 | HS | 0.6162737 | HS | 0.6469837 |
| CIC | 0.52389375 | CIC | 0.5035037 | CIC | 0.5397125 |
| MF | 0.38450125 | MF | 0.3690438 | MF | 0.3872762 |
| OLO | 0.56417500 | OLO | 0.5410150 | OLO | 0.5826237 |
| CB | **0.99195750** | CB | **0.9346750** | CB | **0.9576575** |

**Supplementary Table 4.** The results of TNSS sensitivity analysis

| Fixed |  | Random |  | Eliminate small samples |  | Eliminate low quality score | SUCRA |
| --- | --- | --- | --- | --- | --- | --- | --- |
| Treatment | SUCRA | Treatment | SUCRA | Treatment | SUCRA | Treatment | SUCRA |
| PBO | 0.01433295 | PBO | 0.1445625 | PBO | 0.08337386 | PBO | 0.1879034 |
| HOCl | 0.28690114 | HOCl | 0.3301045 | HOCl | 0.29778523 | HOCl | 0.3429864 |
| MF | 0.36267955 | MF | 0.4278159 | MF | 0.42802045 | MF | 0.4176273 |
| HRS | 0.55758182 | HRS | 0.4531795 | HRS | 0.46477727 | HRS | 0.4397727 |
| NC | 0.42285000 | NC | 0.3855420 | NC | 0.37144091 | NC | 0.3856227 |
| E-Qi | 0.70980114 | E-Qi | 0.5971875 | E-Qi | 0.64848409 | E-Qi | 0.5894886 |
| HS | 0.14205000 | HS | 0.3922102 | HS | 0.36180909 | HS | 0.5040250 |
| XAN | **0.81770682** | XAN | **0.6668761** | XAN | **0.72744659** | XAN | **0.6163830** |
| BUD | **0.90908977** | BUD | **0.8803114** | BUD | **0.94310909** | BUD | **0.8333420** |
| RESV | **0.99999773** | RESV | **0.9629182** | RESV | **0.95201705** | RESV | **0.9215273** |
| S0597 | 0.46162727 | S0597 | 0.4143227 | S0597 | 0.41018636 | S0597 | 0.4075693 |
| FLU | 0.31538182 | FLU | 0.3449693 | FLU | 0.31155000 | FLU | 0.3537523 |

## Supplementary Material

**Supplementary Material 1.** Network Bayesian Meta-Analysis Code

R代码

#安装软件包

install.packages("openxlsx")

install.packages("gemtc")

install.packages("rjags")

Sys.setenv(JAGS_HOME="")

#加载程序包

library(openxlsx)

library(rjags)

library(gemtc)

#设置工作目录

setwd("E:\\meta代做\\结果汇报")

#读入文件

contdata <- read.xlsx("48h疼痛.xlsx",sheet = "dt")

conttrt <- read.xlsx("48h疼痛.xlsx", sheet = "trt")

#构建网状结构

contnw<- mtc.network(data = contdata, treatments = conttrt,

description = "cont network")

plot(contnw, use.description = TRUE)

# 绘制网络图，使用描述名称作为标签，调整点的大小和形状

plot(contnw,

use.description = TRUE, # 显示分类标签的名称

vertex.label.cex = 1, # 网状的点标签大小设定

vertex.label.color = "black", #节点对应标签的颜色

vertex.label.dist=3,#标签的位置

vertex.label.degree=-pi/3, #标签绕点的旋转角度

vertex.color="blue",# 节点的颜色

dynamic.edge.width=TRUE,# 当设置为TRUE时，边的宽度会根据边的属性（如权重）动态调整。这在可视化网络中不同边的重要性时非常有用

edge.color="black",#边的颜色

vertex.label.font=2)#顶点标签的字体类型，1 可能代表默认字体。2 可能代表另一种字体样式，如粗体、斜体或下划线等)

#建立模型

contmdl <- mtc.model(contnw, type = "consistency", factor = 2.5, n.chain = 4,

likelihood = "normal", link = "identity", linearModel = "fixed")

#运算

contrslt<- mtc.run(contmdl, n.adapt = 5000, n.iter = 20000, thin = 1)

summary(contrslt)

#收敛诊断—运算质量检验

gelman.diag(contrslt)

gelman.plot(contrslt)

#相对效应森林图绘制

forest(relative.effect(contrslt, t1 = 1), use.description = TRUE) #以第一行为干预措施，结果描述为标签#

#概率排序图

contrk <- rank.probability(contrslt, preferredDirection = -1) #(结果效应值越小越好,越大越好改为1)

plot(contrk, beside = TRUE)

plot(contrk)

print(contrk)

write.xlsx(contrk, "DBP-RANK.xlsx")

#累计概率排序

contscrk <- gemtc::sucra(contrk)

print(contscrk)

#联赛表生成，查看置信区间跨0是阳性

conttb <- relative.effect.table(contrslt)

contltb <- round(conttb, 2)

write.xlsx(contltb, "K-lian.xlsx")

#不一致性假设的检验-不一致性检验

contns <- mtc.nodesplit(contnw, comparisons = mtc.nodesplit.comparisons(contnw),

likelihood = "normal", link = "identity", linearModel = "fixed")

plot(summary(contns)) #没有闭合环做不了不一致性检验

#同质性检验假设的检验-异质性检验

contht<- mtc.anohe(contnw, likelihood = "normal", link = "identity", linearModel = "fixed")

plot(summary(contht))

**Supplementary Material 2.** Evaluation of Overall Effects and Model Fit

Refer to the attached compressed file.
