# Supplementary figures and images for: Efficacy of different nasal irrigation treatments versus placebo in allergic rhinitis: a systematic review and network meta-analysis
Source: Front Pharmacol. 2025 Nov 10;16:1670372. doi: 10.3389/fphar.2025.1670372 (PMC12641607; doi:10.3389/fphar.2025.1670372)

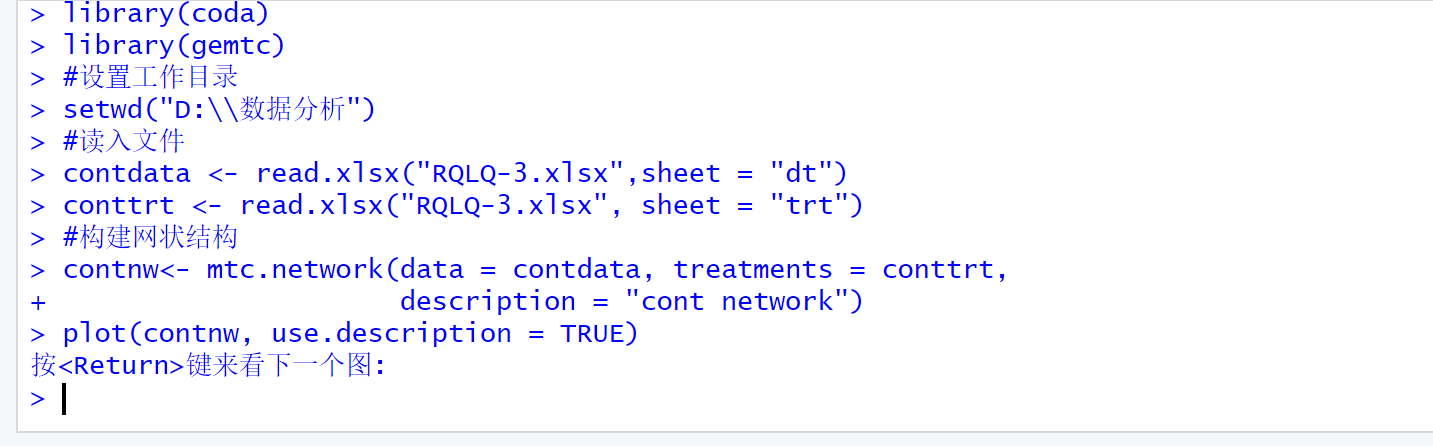

Supplement: Supplementary file 2 [file DataSheet1.zip › RQLQ/RQLQ network diagram1.tiff]

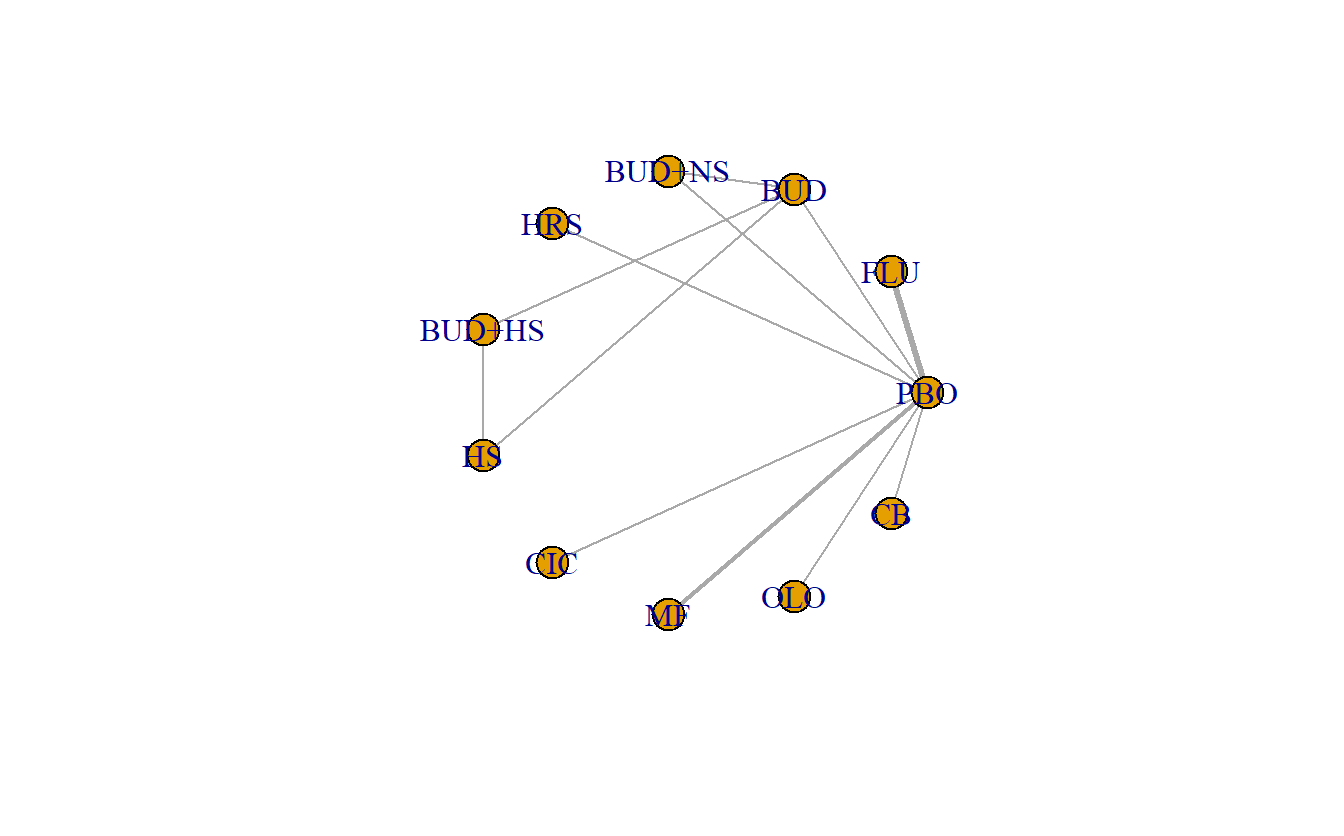

Supplement: Supplementary file 2 [file DataSheet1.zip › RQLQ/RQLQ network diagram2.tiff]

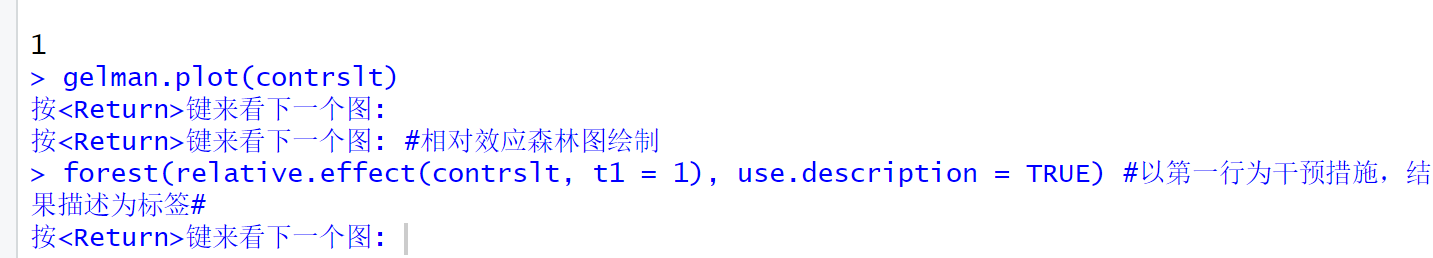

Supplement: Supplementary file 2 [file DataSheet1.zip › RQLQ/RQLQ forest plot1.tiff]

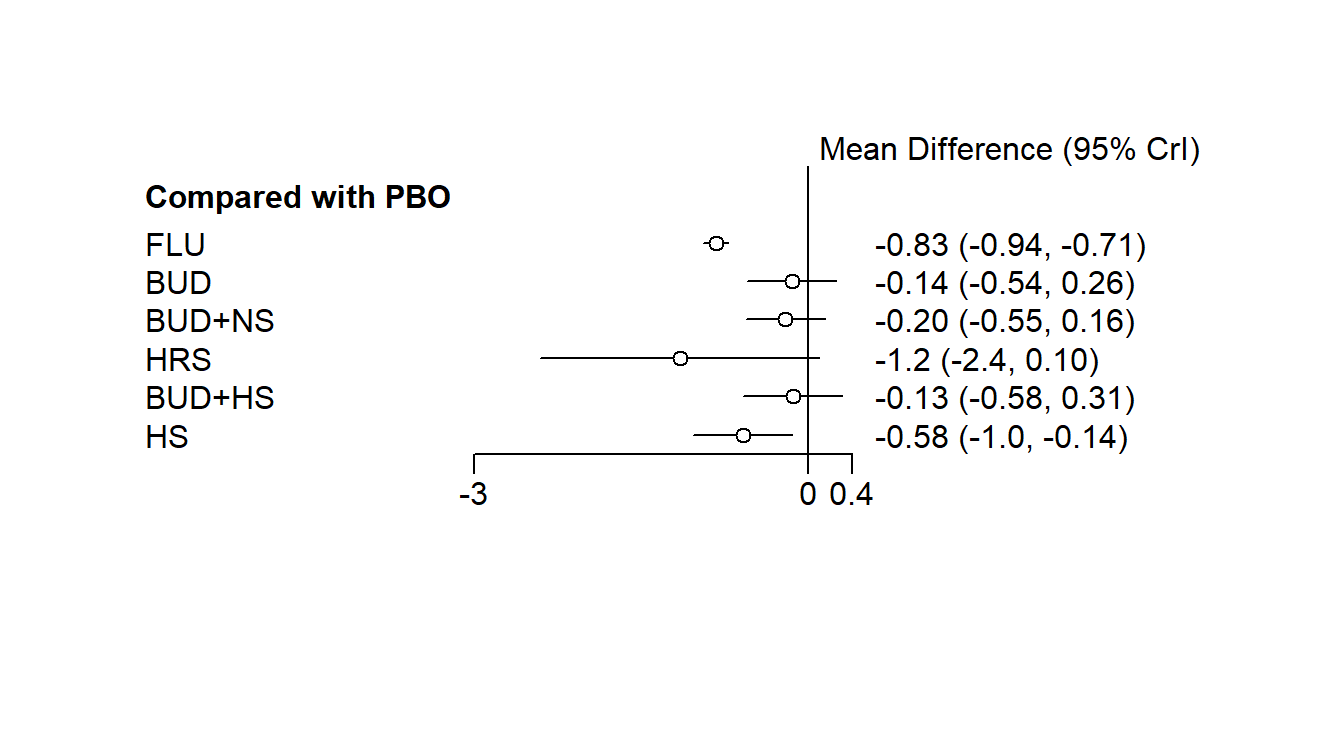

Supplement: Supplementary file 2 [file DataSheet1.zip › RQLQ/RQLQ forest plot2.tiff]

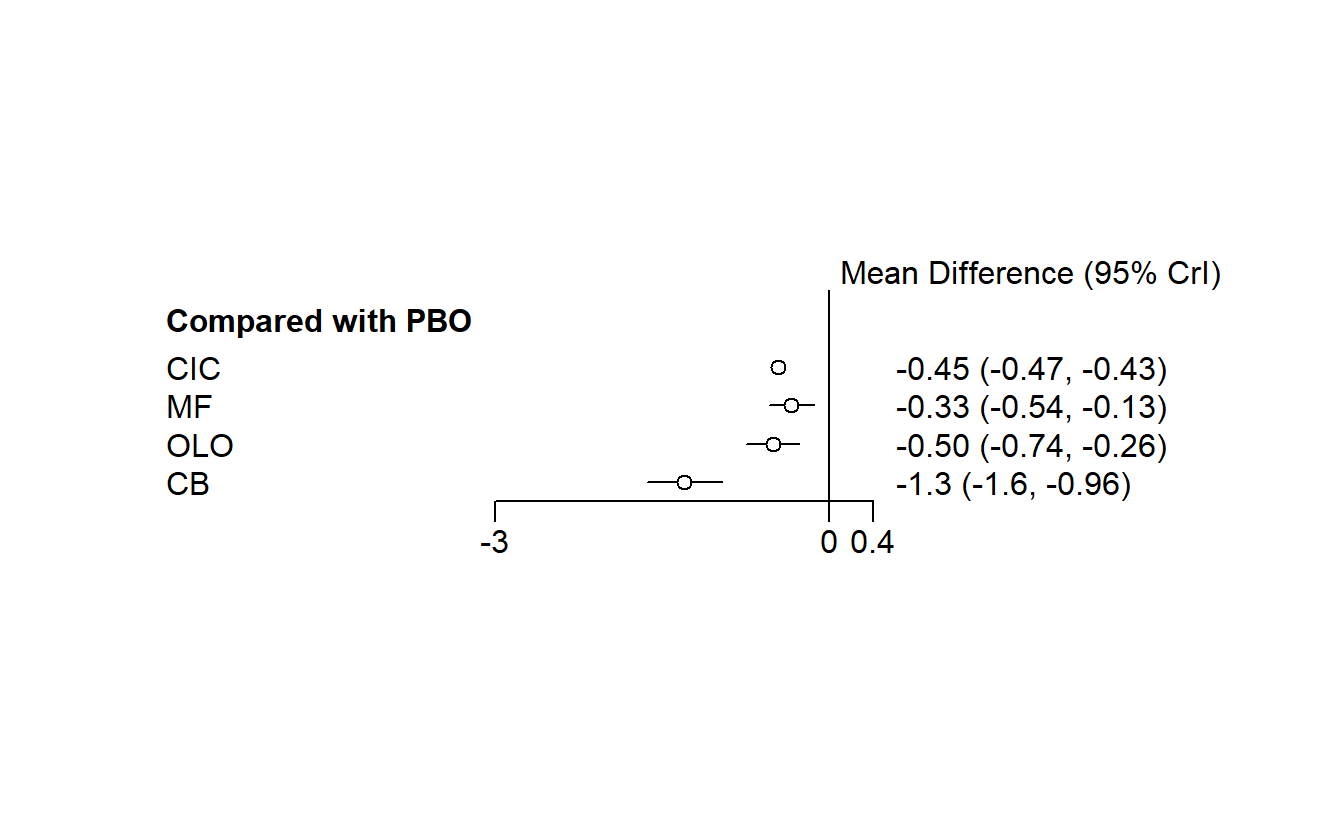

Supplement: Supplementary file 2 [file DataSheet1.zip › RQLQ/RQLQ forest plot3.tiff]

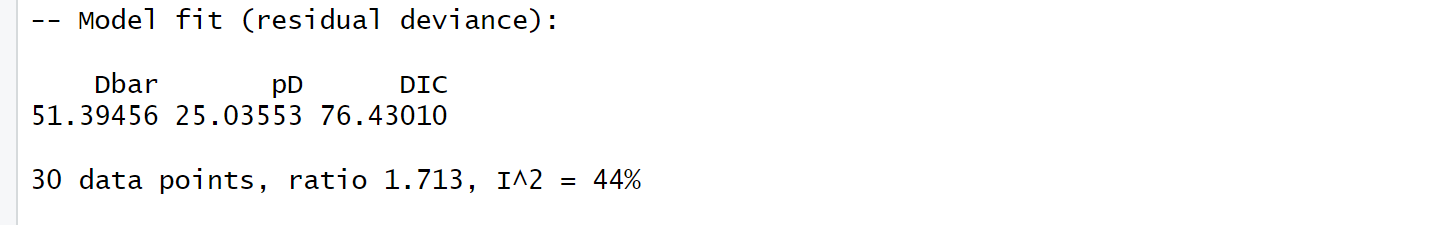

Supplement: Supplementary file 2 [file DataSheet1.zip › RQLQ/RQLQ I^2.tiff]

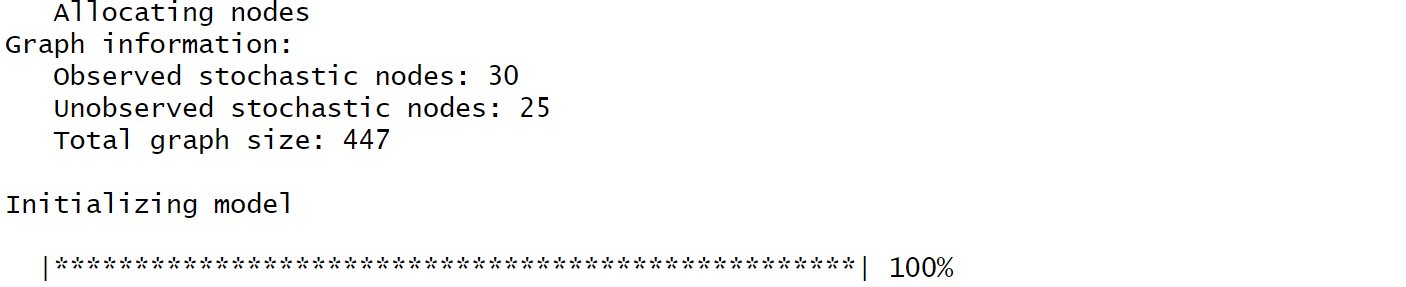

Supplement: Supplementary file 2 [file DataSheet1.zip › RQLQ/RQLQ Inconsistency Test1.tiff]

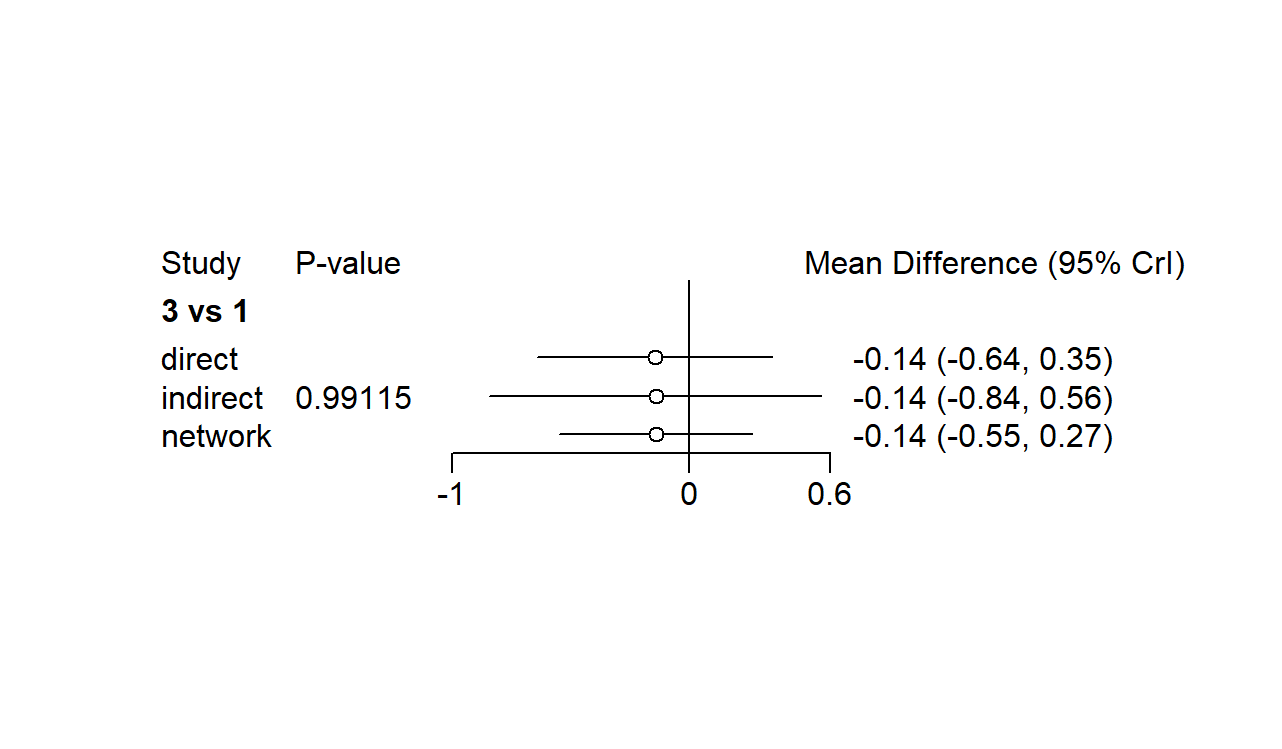

Supplement: Supplementary file 2 [file DataSheet1.zip › RQLQ/RQLQ Inconsistency Test2.tiff]

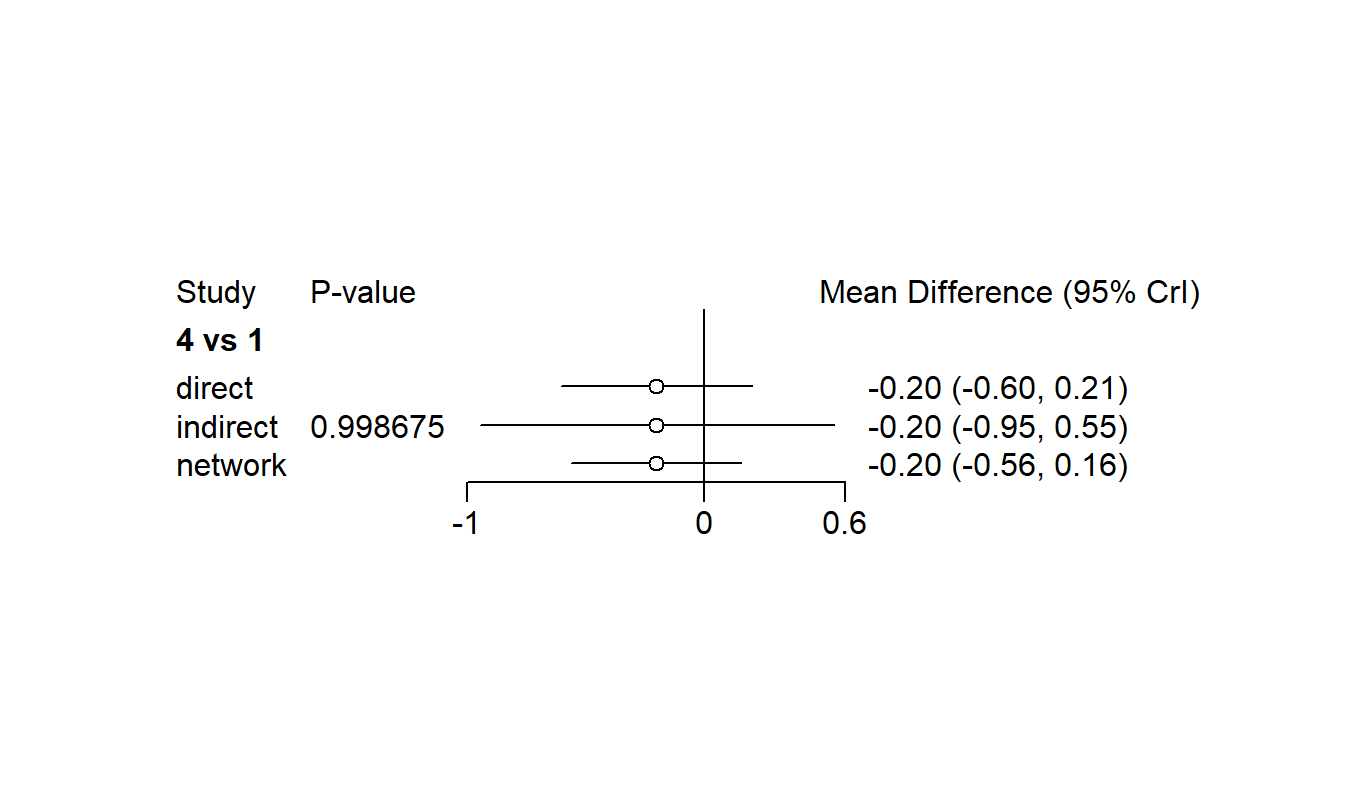

Supplement: Supplementary file 2 [file DataSheet1.zip › RQLQ/RQLQ Inconsistency Test3.tiff]

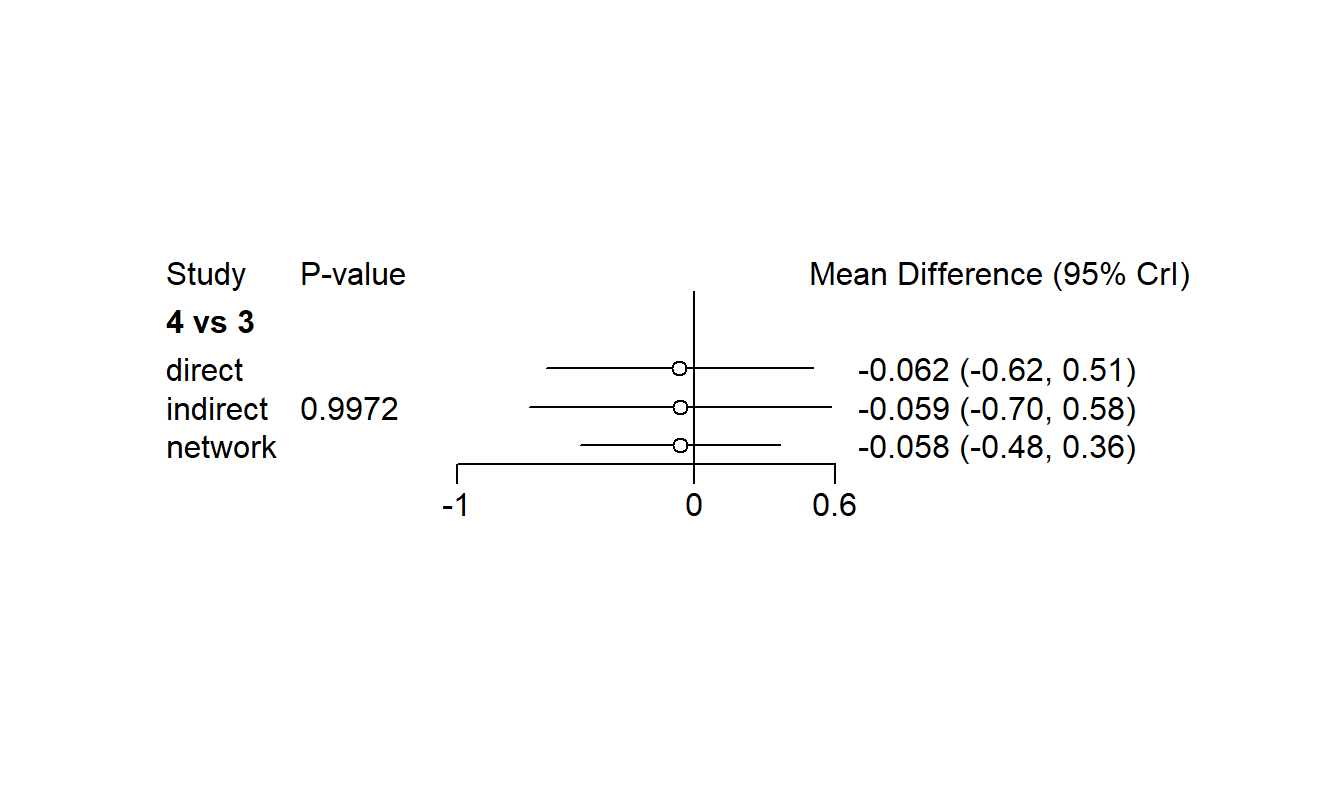

Supplement: Supplementary file 2 [file DataSheet1.zip › RQLQ/RQLQ Inconsistency Test4.tiff]

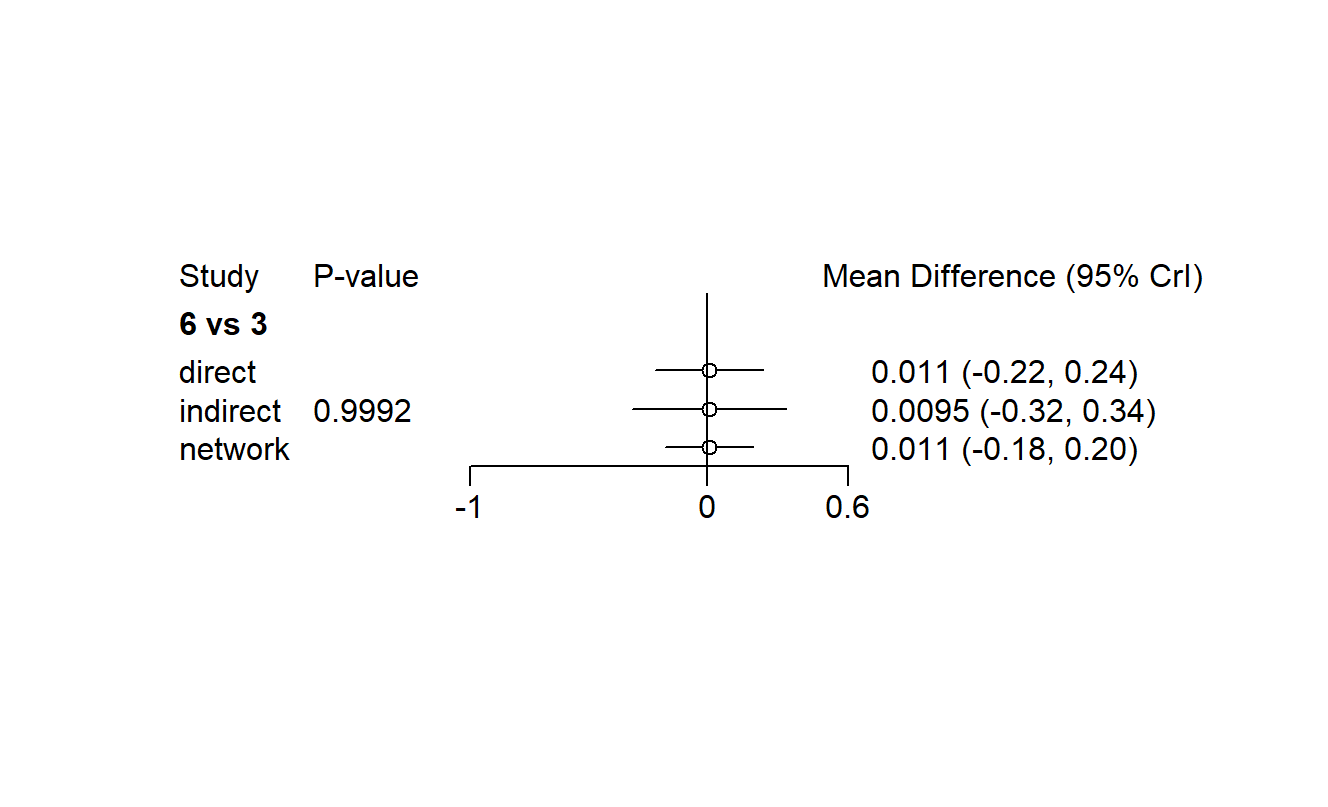

Supplement: Supplementary file 2 [file DataSheet1.zip › RQLQ/RQLQ Inconsistency Test5.tiff]

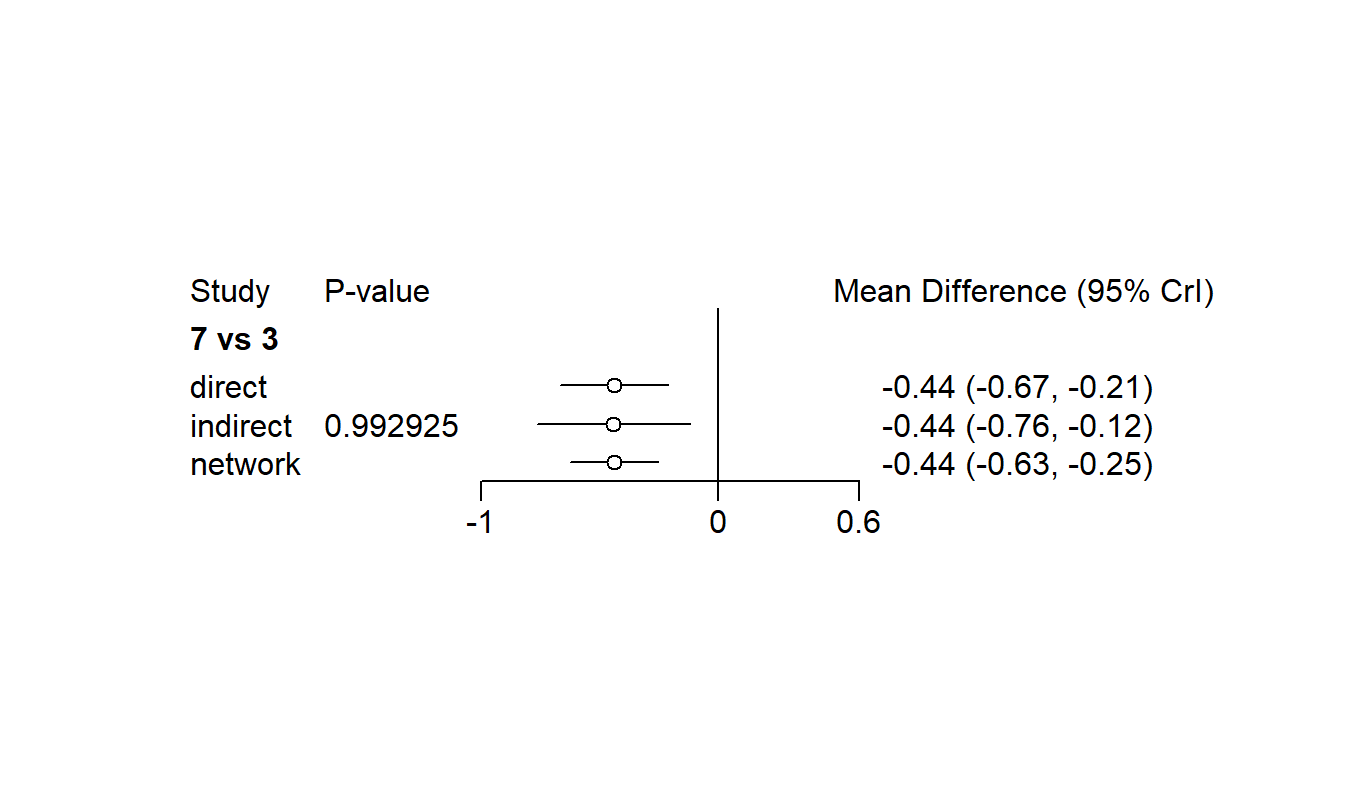

Supplement: Supplementary file 2 [file DataSheet1.zip › RQLQ/RQLQ Inconsistency Test6.tiff]

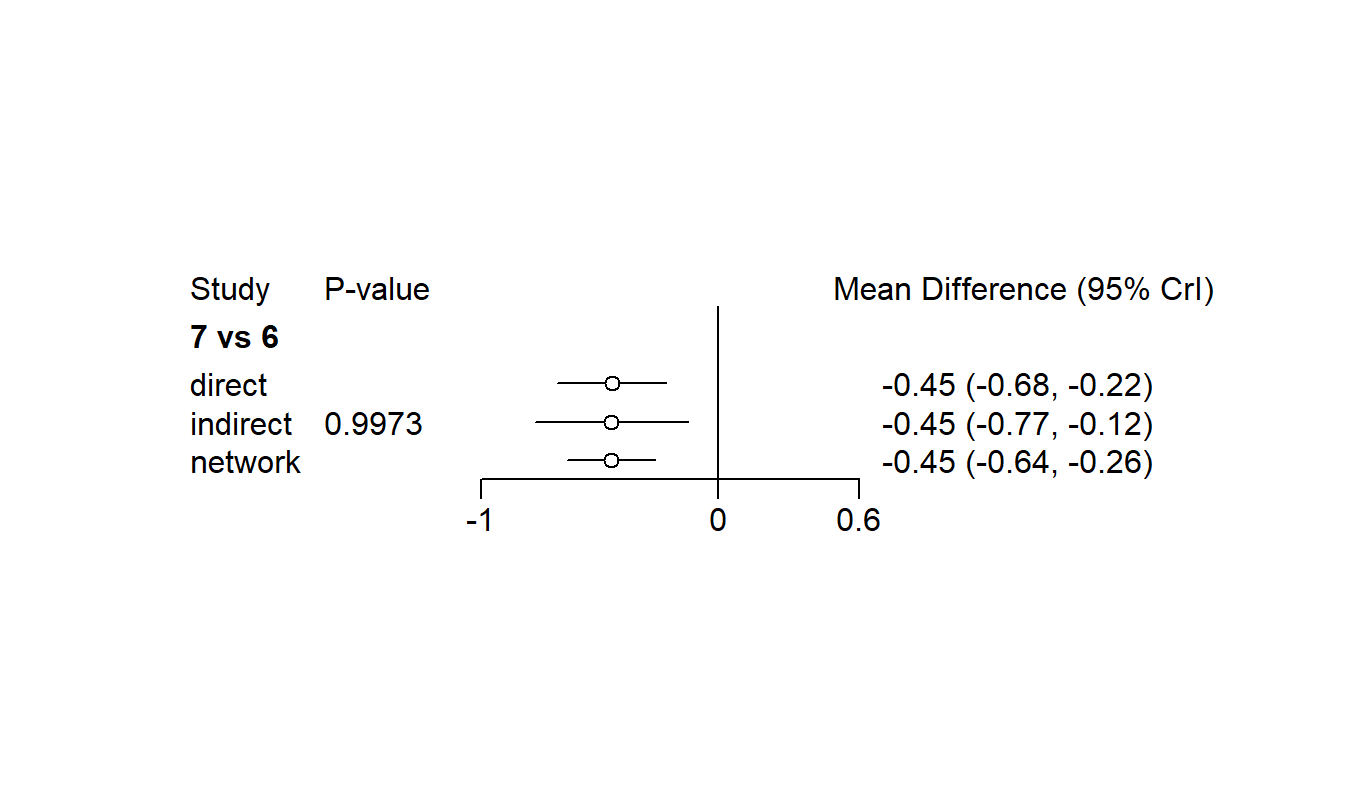

Supplement: Supplementary file 2 [file DataSheet1.zip › RQLQ/RQLQ Inconsistency Test7.tiff]

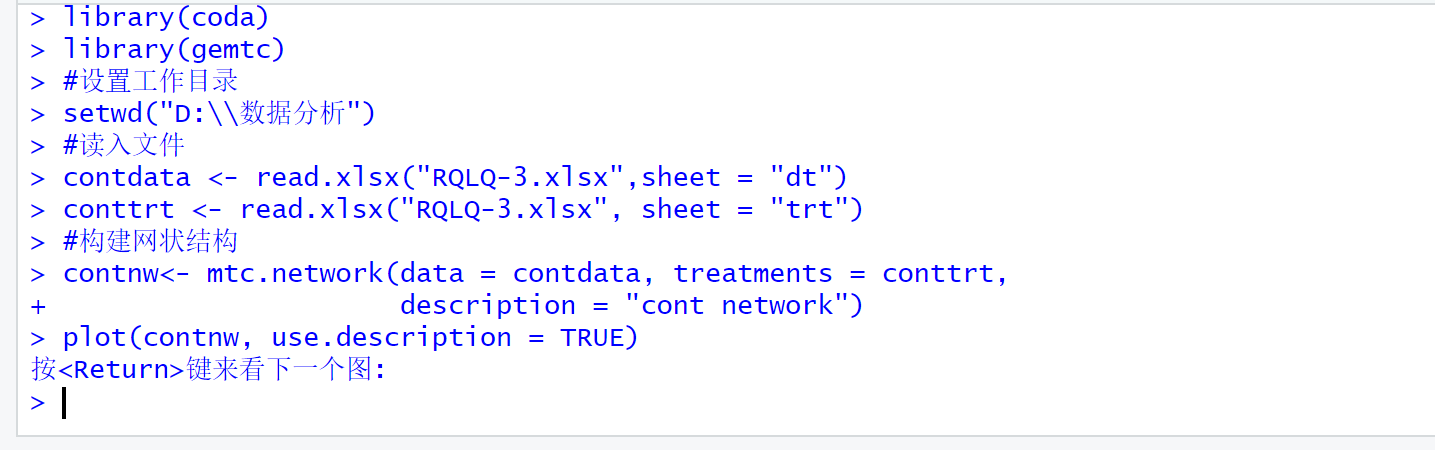

Supplement: Supplementary file 2 [file DataSheet1.zip › RQLQ/RQLQ network diagram3.tiff]

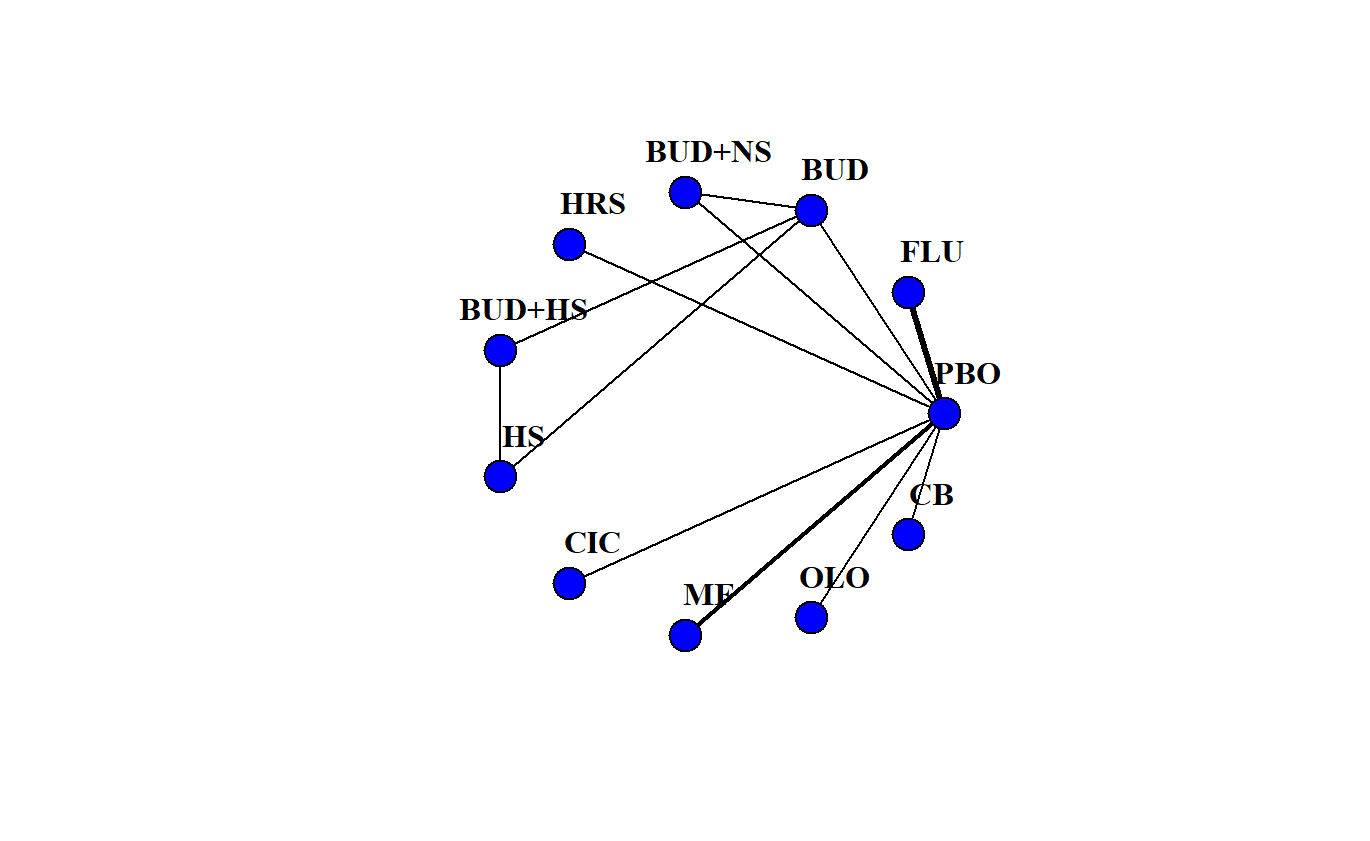

Supplement: Supplementary file 2 [file DataSheet1.zip › RQLQ/RQLQ network diagram4.tiff]

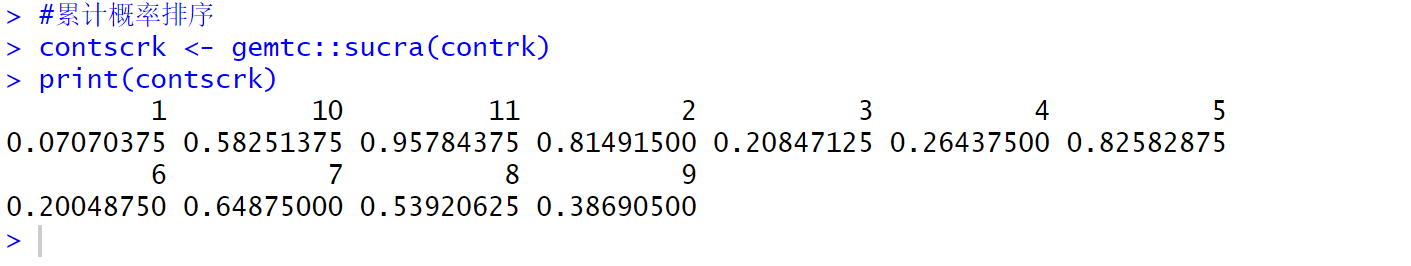

Supplement: Supplementary file 2 [file DataSheet1.zip › RQLQ/RQLQ SUCRA.tiff]

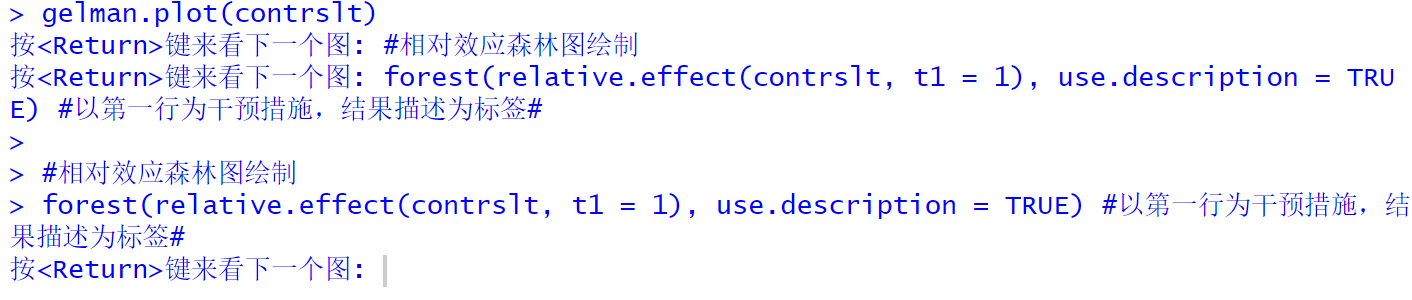

Supplement: Supplementary file 2 [file DataSheet1.zip › TNSS/TNSS forest plot1.tiff]

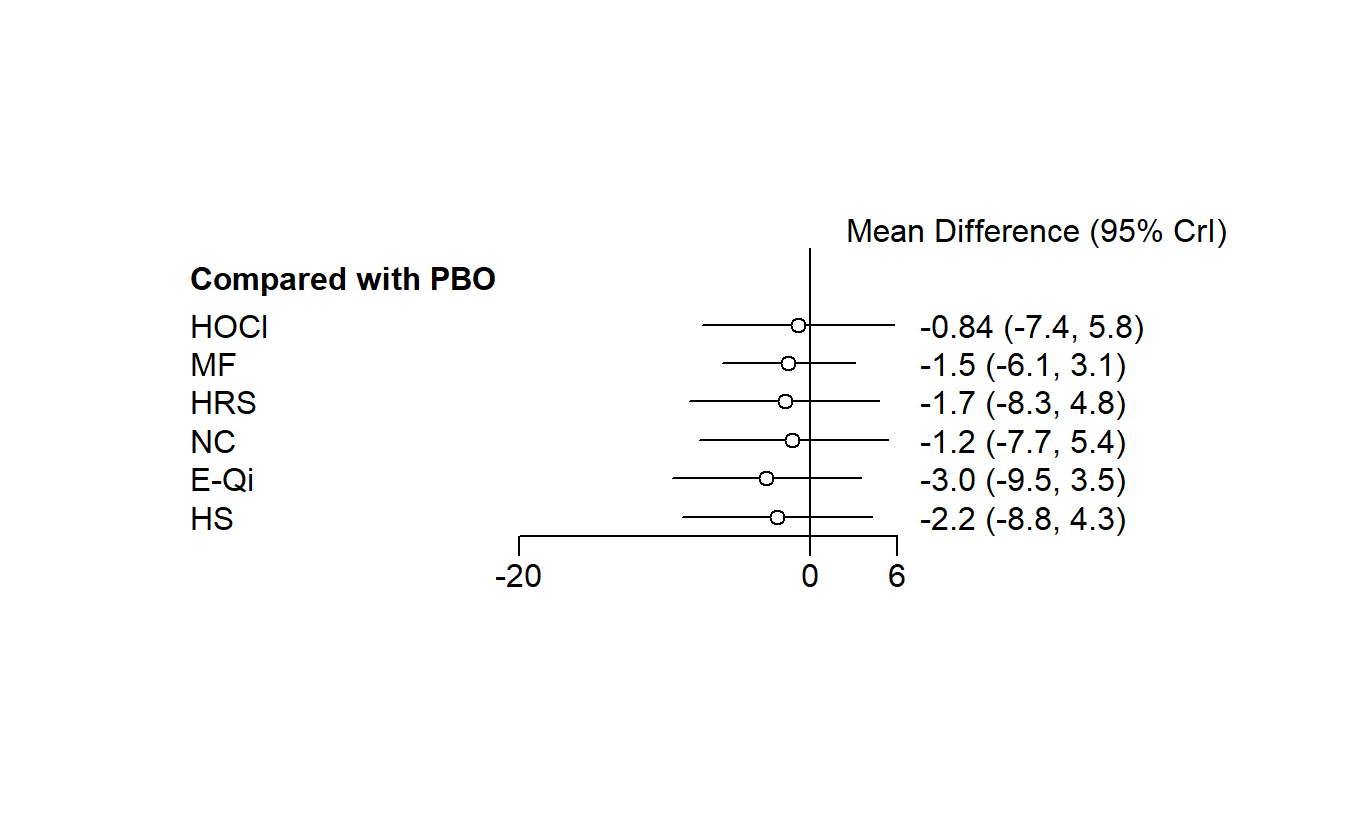

Supplement: Supplementary file 2 [file DataSheet1.zip › TNSS/TNSS forest plot2.tiff]

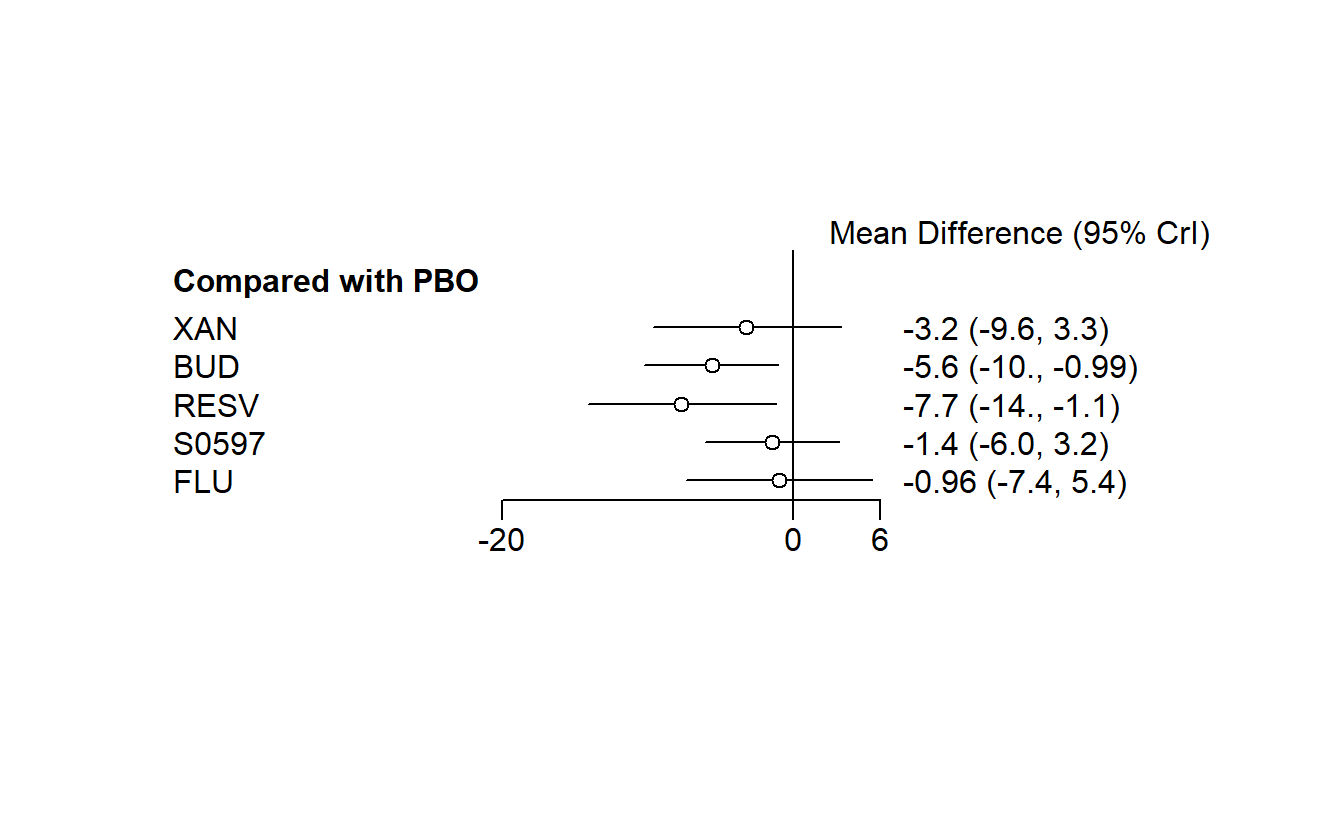

Supplement: Supplementary file 2 [file DataSheet1.zip › TNSS/TNSS forest plot3.tiff]

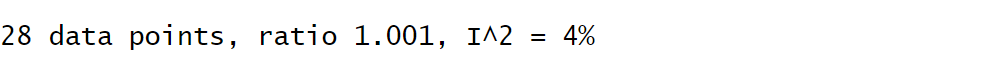

Supplement: Supplementary file 2 [file DataSheet1.zip › TNSS/TNSS I^2.tiff]

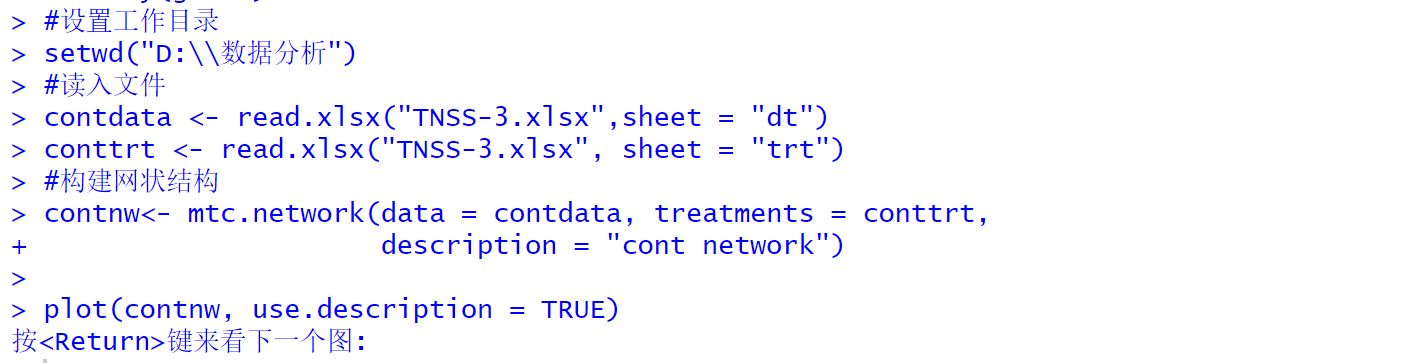

Supplement: Supplementary file 2 [file DataSheet1.zip › TNSS/TNSS network diagram.tiff]

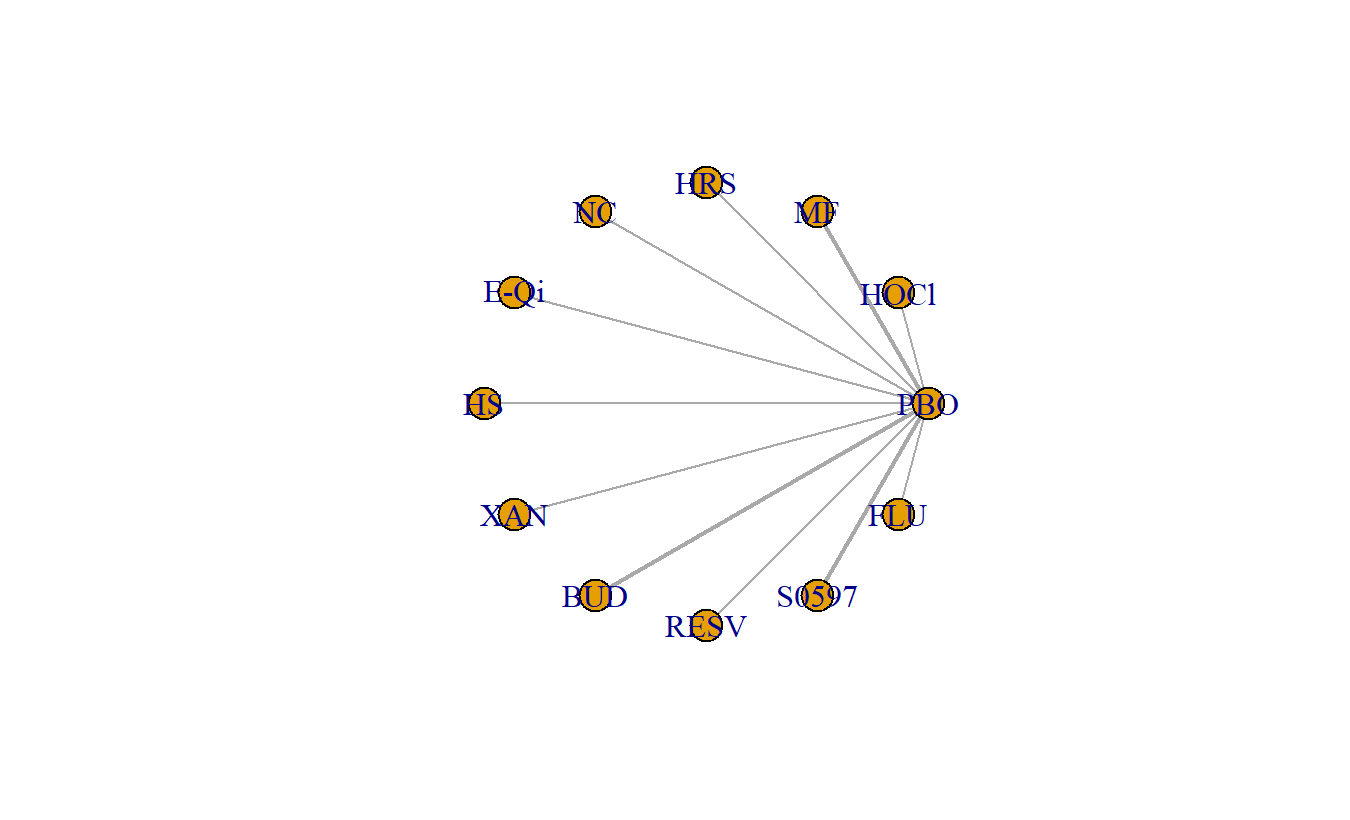

Supplement: Supplementary file 2 [file DataSheet1.zip › TNSS/TNSS network diagram2.tiff]

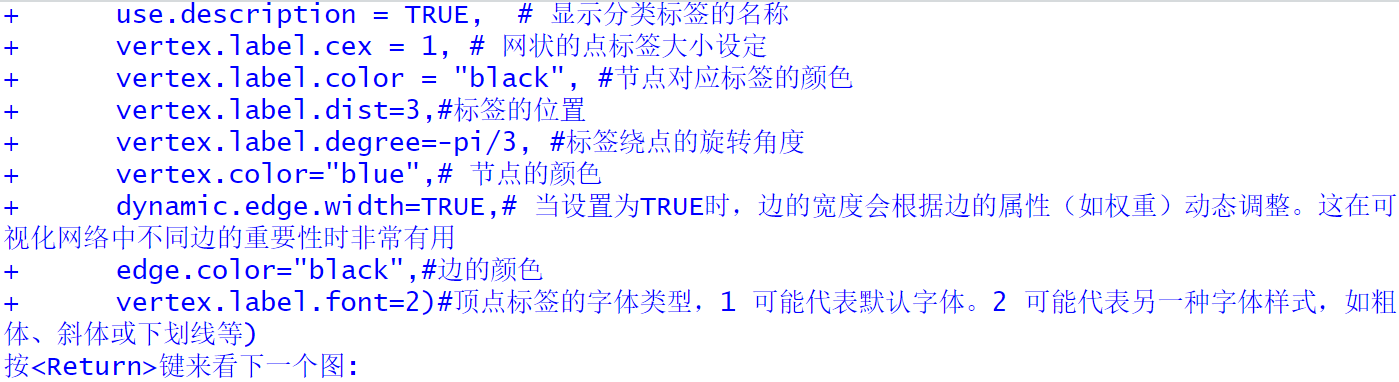

Supplement: Supplementary file 2 [file DataSheet1.zip › TNSS/TNSS network diagram3.tiff]

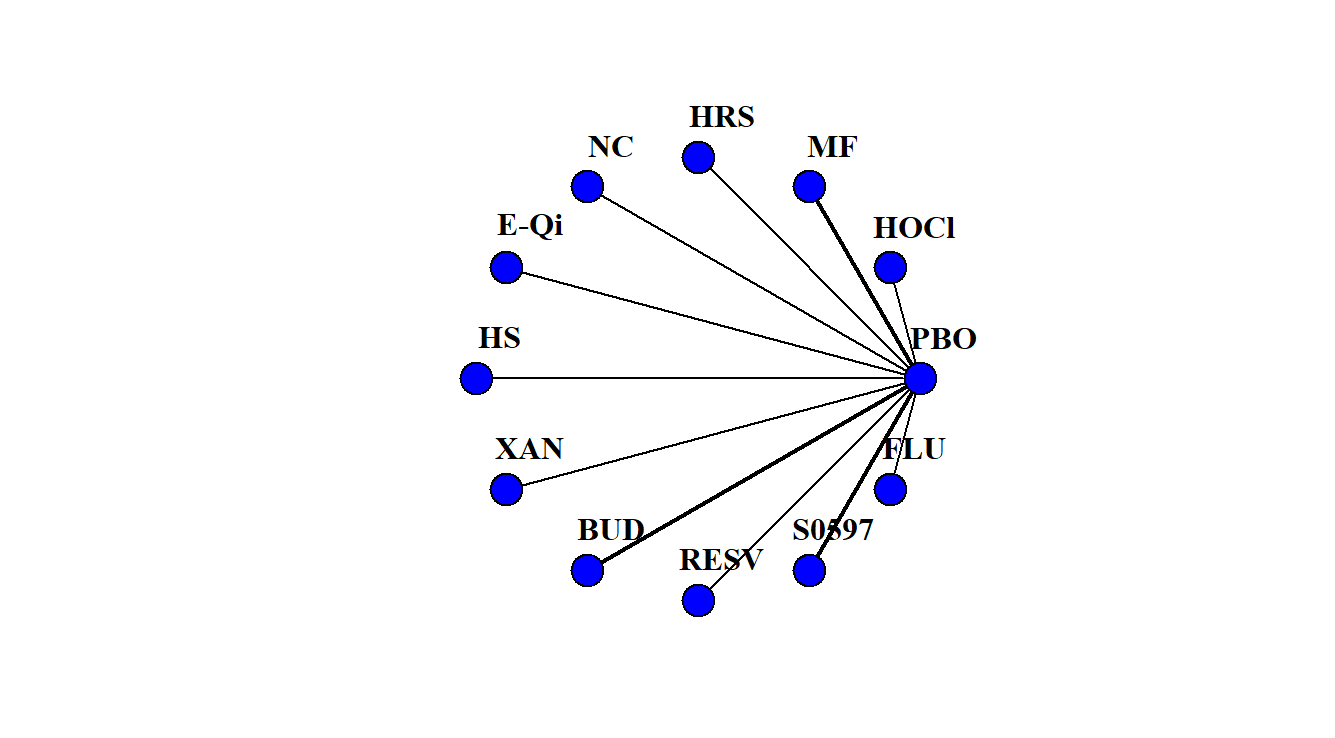

Supplement: Supplementary file 2 [file DataSheet1.zip › TNSS/TNSS network diagram4.tiff]

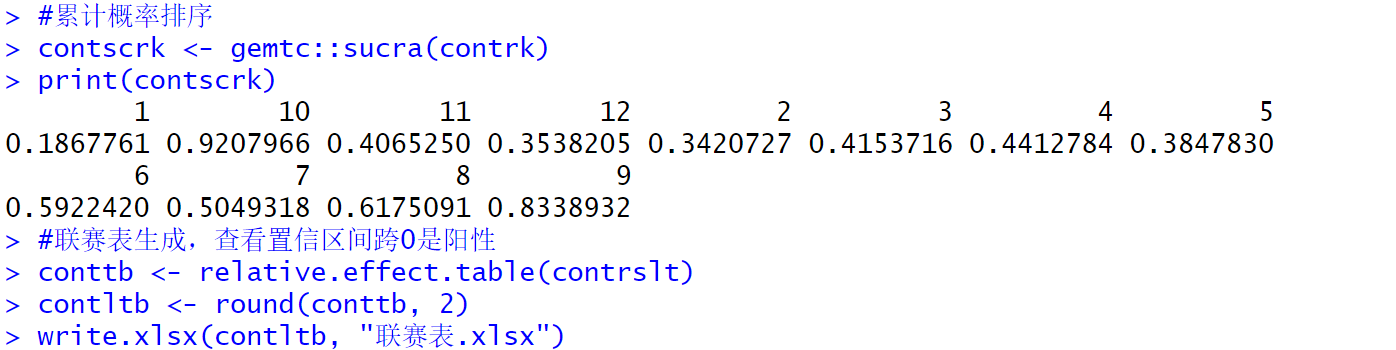

Supplement: Supplementary file 2 [file DataSheet1.zip › TNSS/TNSS SUCRA.tiff]
